# A highly robust and optimized sequence-based approach for genetic polymorphism discovery and genotyping in large plant populations

**DOI:** 10.1007/s00122-016-2736-9

**Published:** 2016-06-17

**Authors:** Ning Jiang, Fengjun Zhang, Jinhua Wu, Yue Chen, Xiaohua Hu, Ou Fang, Lindsey J. Leach, Di Wang, Zewei Luo

**Affiliations:** 1 Department of Biostatistics and Computational Biology, SKLG, School of Life Sciences, Fudan University, Shanghai, China; 2 School of Biosciences, The University of Birmingham, Edgbaston, Birmingham, B15 2TT UK; 3 Gansu Agricultural University, Lanzhou, Gansu China; 4 Qinghai Academy of Agriculture and Forestry Sciences, Xining, Qinghai China

## Abstract

****Key message**:**

**This optimized approach provides both a computational tool and a library construction protocol, which can maximize the number of genomic sequence reads that uniformly cover a plant genome and minimize the number of sequence reads representing chloroplast DNA and rRNA genes. One can implement the developed computational tool to feasibly design their own RAD-seq experiment to achieve expected coverage of sequence variant markers for large plant populations using information of the genome sequence and ideally, though not necessarily, information of the sequence polymorphism distribution in the genome.**

**Abstract:**

Advent of the next generation sequencing techniques motivates recent interest in developing sequence-based identification and genotyping of genome-wide genetic variants in large populations, with RAD-seq being a typical example. Without taking proper account for the fact that chloroplast and rRNA genes may occupy up to 60 % of the resulting sequence reads, the current RAD-seq design could be very inefficient for plant and crop species. We presented here a generic computational tool to optimize RAD-seq design in any plant species and experimentally tested the optimized design by implementing it to screen for and genotype sequence variants in four plant populations of diploid and autotetraploid *Arabidopsis* and potato *Solanum tuberosum*. Sequence data from the optimized RAD-seq experiments shows that the undesirable chloroplast and rRNA contributed sequence reads can be controlled at 3–10 %. Additionally, the optimized RAD-seq method enables pre-design of the required uniformity and density in coverage of the high quality sequence polymorphic markers over the genome of interest and genotyping of large plant or crop populations at a competitive cost in comparison to other mainstream rivals in the literature.

**Electronic supplementary material:**

The online version of this article (doi:10.1007/s00122-016-2736-9) contains supplementary material, which is available to authorized users.

## Introduction

Simultaneously identifying genetic variants and genotyping across the whole genome in large populations of interest has become one of the primary objectives of genomic studies and has been widely carried out for linkage analysis, association mapping, marker assisted selection, ecological and evolutionary studies (Davey et al. 2011; Gonen et al. 2014; Gray et al. 2000; Luikart et al. 2003; Poland and Rife 2012). With the advent of the Next Generation Sequencing (NGS) technologies, many sequence-based genotyping approaches have been developed (Bahassi and Stambrook 2014; Davey and Blaxter 2010; Metzker 2010; Varshney et al. 2009). The original approach was DNA-seq, which involves sequencing the whole genome for complete identification of all genome-wide genetic variants (Shendure and Ji 2008). Although the cost of NGS has continuously declined over the past decade, it is still unfeasible to carry out whole genome DNA sequencing in large populations of most eukaryotic species, which generally have large genome sizes, from many millions to even billions of base pairs (Rowe et al. 2011; Wetterstrand 2012).

Recently, several ‘reduced-representation’ sequencing approaches have been developed to address the cost limitations of DNA-seq. For example, Chepelev et al., Geraldes et al. and Hamilton et al. have all used RNA-sequencing to identify tens to thousands of genetic variants in human, cottonwood and potato genomes (Chepelev et al. 2009; Geraldes et al. 2011; Hamilton et al. 2011). By focusing only on the transcriptome, the amount of short read data required and associated costs are dramatically reduced. However, the expression levels of genes across samples can vary widely and change dynamically, bringing various challenges for mining genotypic information from transcriptome data (Christodoulou et al. 2011; Nothnagel et al. 2011). To avoid these challenges, hybrid capture sequencing strategies have been developed (Kiialainen et al. 2011; Mamanova et al. 2010). When the nucleotide sequence information of regions of interest are known, they can be directly extracted and sequenced using specific hybrid-capture probes (Clark et al. 2011). Recently, Uitdewilligen et al. designed 57,054 oligonucleotide probes to capture DNA fragments for sequencing 807 potato genes and successfully inferred sample genotypes from the sequence data (Uitdewilligen et al. 2013). An obvious technical hurdle to widespread use of this hybrid capture sequencing strategy is the requirement for comprehensive genomic sequence information to enable design of a large number of unique capture probes for genotyping predefined genome regions. Furthermore, the extracted DNA samples may not evenly cover the targeted genomic regions. For instance, the extracted DNA is generally enriched in targeted regions with high GC content, due to more stable binding (Mertes et al. 2011).

In 2008, Baird et al. was the first to implement a Restriction site Associated DNA (RAD) approach with NGS for cost effective genome-wide identification and genotyping of DNA sequence variants (Baird et al. 2008). The basic idea behind the RAD-seq approach was to use a restriction enzyme (RE) to cut genomic DNA into fragments, and collect only those fragments with specified lengths for sequence library construction followed by sequencing. This led to the development of a number of other strategies including Genotyping-by-Sequencing, or GBS (Elshire et al. 2011; Sonah et al. 2013), and 2-enzyme GBS (Poland et al. 2012a). In 2012, three other groups (Peterson et al. 2012; Poland et al. 2012b; Truong et al. 2012) developed methods to extend the original RAD-seq approach to include double digestion (ddRAD-seq) combining two REs to produce RAD-seq libraries consisting of a barcoded adapter and a universal adapter on two ends of each selected DNA fragment. The use of two REs in RAD-seq (or GBS) enabled more flexibility in the choice of sequenced regions across the target genome and has been widely used in plant species (Chen et al. 2013; Poland and Rife 2012). An alternative approach called 2b-RAD was developed by Wang et al. (2012b), which uses type IIB restriction enzymes (BsaXI or AlfI) allowing the target genome to be sheared into DNA fragments with a constant length of 33 bp (BsaXI) or 36 bp (Alfl) for the identification of genetic variants within these short DNA fragments. Further improvements to simplify and reduce costs of the original RAD-seq protocol have also been developed, including ezRAD-seq (Toonen et al. 2013).

Applying reduced-representation sequencing approaches (especially RAD-seq) to plant species has brought new and significant challenges that have not been adequately addressed by existing approaches. Firstly, a large proportion (30–60 %) of obtained short reads may be mapped to chloroplast sequence and rRNA genes (Chen et al. 2013; Romero-Severson et al. 2014; Truong et al. 2012; Wang et al. 2012b), due to the large number of copies of chloroplast (1000–10,000) and rRNA gene DNA sequences per cell in plant leaf tissue (Bendich 1987; Wang et al. 2009). This represents a serious waste of high-throughput sequencing capacity and resources since these reads are typically directly discarded for downstream analysis (Chen et al. 2013; Romero-Severson et al. 2014; Truong et al. 2012; Wang et al. 2012b). Secondly, it is crucial to achieve high recovery of DNA fragments within a specified size range during the size-selection step. However, existing RAD-seq approaches have generally implemented traditional manual gel purification to extract the targeted DNA fragments (Baird et al. 2008; Chen et al. 2013; Chutimanitsakun et al. 2011; Etter et al. 2011; Pfender et al. 2011; Wang et al. 2013). This method is imprecise, has low reproducibility, and can introduce low molecular weight contamination (Life and Technologies 2015). Peterson et al. introduced the use of Pippin-Prep automated size-selection technology to precisely extract the targeted DNA fragments (Peterson et al. 2012).

We report here a new optimized RAD-seq approach for identifying and genotyping polymorphic genetic markers in large plant populations. The optimization was made to maximize the number of genomic DNA sequence reads that uniformly cover a plant genome under study and minimize the number of sequence reads representing chloroplast DNA and rRNA genes. The optimized approach provides both a computational tool and a library construction protocol. The computational pipeline was developed in a user friendly manner to work out the possible DNA segments to be generated from cutting a given plant genome of interest by using different combinations of REs, and then to determine the optimal combination of REs. The computational tools and the corresponding computer programs were generic in design and compilation, enabling their use in the design of RAD-seq experiments without needing specialized bioinformatics or computational skills. Experimentally, we proposed here a double size-selection strategy using the Pippin Prep automated size selection technology, to reproducibly extract DNA fragments with a pre-defined size range from the computational prediction, and at the same time, to enable removal of dimers from the RAD-seq libraries (Life and Technologies 2015; Quail et al. 2008). To experimentally validate the optimized RAD-seq approach, we constructed 6 pooled sequencing libraries for parental lines and their segregating offspring of both diploid and tetraploid *Arabidopsis* and potato (*Solanum tuberosum*). The sequence data show that the optimized RAD-seq approach designed for the *Arabidopsis* and potato genomes can effectively remove DNA fragments derived from chloroplast sequence and rRNA genes, and the short reads collected are mostly concentrated onto the targeted genomic regions. A balanced representation of sequence reads was obtained from across all pooled samples. These features confirm the robustness and efficiency of the optimized RAD-seq approach developed here and further indicate that one can feasibly design and effectively implement the protocol to achieve expected coverage of polymorphic sequence markers for large plant populations given information of the genome sequence and ideally, though not necessarily, information of the sequence polymorphism distribution in the genome.

## Materials and methods

### Bioinformatic analysis for design of an optimized RAD-seq experiment

We proposed here an optimized RAD-seq approach for identifying and genotyping genetic polymorphic markers in large plant populations, which differs in two main ways from existing RAD-seq protocols in the literature. Specifically, we developed computer simulation, which was programed in FORTRAN-90, to carry out two rounds of digestion with different combinations of restriction enzymes (REs) for a given plant genome of interest. The simulation requires sequence information of the genome and information of the cutting sites of all possible REs. These two rounds of RE digestion are designed to not only cut genomic DNA sequence into pre-designed fragments but also to remove the large proportion of DNA fragments from chloroplast sequence and rRNA genes during RAD-seq library construction.

Although generic for any plant genome, the bioinformatic analysis was illustrated here with a model plant (*Arabidopsis*) and a crop plant (potato, *Solanum tuberosum*). The genomic and chloroplast sequences of *Arabidopsis* and potato were downloaded from The *Arabidopsis* Information Resource (TAIR, https://www.Arabidopsis.org/download/index.jsp) and the potato Genome Sequencing Consortium (PGSC, http://potatogenomics.plantbiology.msu.edu). For the first round of in silico digestion, we selected 4 widely used REs (EcoRI, HindIII, MspI and MseI) to cut the *Arabidopsis* or potato genomic sequence to select the optimal combination of REs based on the following criteria: (1) recovery of the largest possible number of genomic regions within the target size range required by Illumina sequencing (224–424 bp). This number should be at least as large as the predefined number (here, 10,000 regions) based on consideration of the experimental objectives, including the coverage required per sample, the number of samples to be sequenced, and the density of DNA polymorphisms to be targeted; 2) minimization of the number of DNA fragments recovered from chloroplast sequence and rRNA genes. For the second round of digestion, we employed the 269 commercially available REs (http://insilico.ehu.es/restriction/main/index2.php) to achieve the optimal combination of REs for completely removing the DNA fragments from chloroplast sequence and rRNA genes while keeping the maximum number of selected genomic DNA fragments intact. The computer programs developed here search and compare exhaustively the DNA segments to be generated by all possible combinations of enzymes against the optimal criteria (1) and (2) aforementioned, and will print out the optima for selection. The programs can be found at http://www.statisticalgenetics.info/software.html. The second part of the optimized RAD-seq strategy involved the introduction of a double size-selection strategy using the Pippin-Prep automated size selection technology for precise selection of targeted DNA fragments (see S1 Protocol for detailed information).

### Plant samples and DNA collection

Two *Arabidopsisthaliana* parental ecotypes, Columbia (Col) and Landsberg (Ler), were crossed to generate F1 offspring. The F1 offspring plants were randomly mated to produce segregating F2 populations. A tetraploid F2 population was produced similarly using isogenic and artificially induced and selected autotetraploid parental lines of Col (ABRC; CS3151) and Ler (ABRC; CS3900), which supplied by the Arabidopsis Biological Resource Center (ABRC, http://abrc.osu.edu/). For both diploid and tetraploid populations, the sequenced samples consisted of 10 F2 segregating offspring plants and two parental lines. Independently, a diploid potato F1 population was produced by crossing two highly heterozygous ecotypes BD6-6 and BD66-6 originating from China. A tetraploid potato F1 population was produced by crossing the highly heterozygous ecotype Atlantic from the USA with the heterozygous ecotype Longshu-3 originating from China. The population used for sequencing consisted of 10 F1 offspring plants and two parental lines for both diploid and tetraploid varieties. Total DNA was extracted from leaf tissue using the DNeasy Plant Mini Kit according to the manufacture’s procedures (QIAGEN, Valencia, CA, USA).

### Barcoded sequencing library construction

**Fig. 1:**
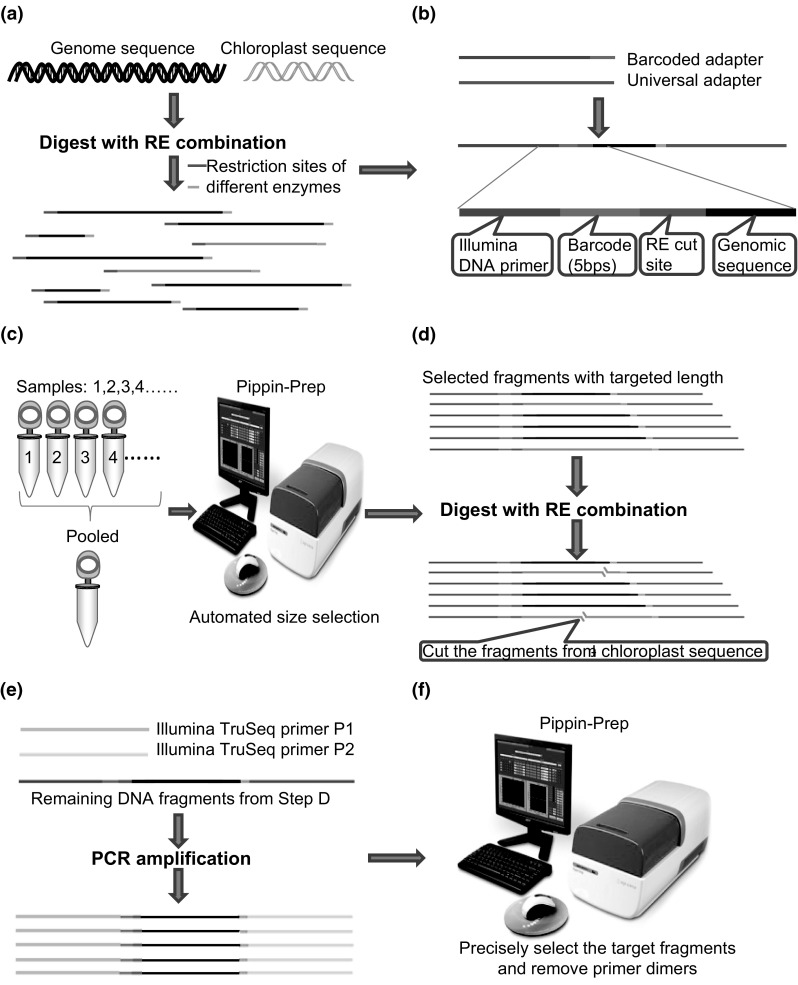
Workflow of modified RAD-seq library construction. **a** shearing the cellular DNA into fragments, **b** ligating the adapters to fragment ends, **c** pooling of samples and fragment size selection, **d** second round of digestion to remove the DNA fragments from rRNA genes and chloroplast sequence, **e** PCR amplification, **f** second round of fragment size selection

The workflow of library construction for the optimized RAD-seq protocol was shown in Fig. 1. In brief, it consists of the following 6 steps: (1) first round of digestion to cut the DNA sequence of each sample; (2) ligating the barcoded adapters to RE cut sites; (3) pooling equimolar amounts of DNA fragments for each sample and carrying out precise size-selection using the Pippin Prep automated size selection platform; (4) second round of digestion to remove the selected DNA fragments from chloroplast sequence and rRNA genes; (5) PCR amplification using Illumina primers for pooled samples; (6) second round of size-selection using Pippin Prep to ensure high recovery of DNA fragments in the required size range and remove adapter dimers.

For the pooled library construction strategy, dozens of modified Illumina adapters were designed for different RE cut sites: for the MspI cut site ligation, a universal adapter attached with Biotin was designed (top oligo: 5′-Biotin-GTGACTGGAGTTCAGACGTGTGCTCTTCCGATCT-3′; bottom oligo: 5′-CGAGATCGGAAGAGCGAGAACAA-3′); for the EcoRI cut site ligation, barcoded (sample-specific) adapters were designed (top oligo: 5′-ACACTCTTTCCCTACACGACGCTCTTCC GATCTxxxxx-3′; bottom oligo: 5′-AATTxxxxxAGATCGGAAGAGCGTCGTGTAGGGAAAGAGTGT-3′); similarly, for the HindIII cut site ligation, barcoded adapters were designed (top oligo: 5′-ACACTCTTTCC CTACACGACGCTCTTCCGATCTxxxxx-3′; bottom oligo: 5′-AGCTxxxxxAGATCGGAAGAGCGTCGT GTAGGGAAAGAGTGT-3′). The unique 5 bp barcodes (xxxxx) allowed identification of all different samples from the pooled library. A complete list of all modified Illumina adapters used in this study was shown in S1 Table.

### Illumina sequencing and read mapping

For *Arabidopsis* pooled libraries, 2 × 150 bp paired-end sequencing was performed on the Illumina MiSeq platform. For potato pooled libraries, 2 × 100 bp paired-end sequencing was performed using the Illumina HiSeq 2000 platform. A FORTRAN program was developed to allocate the short reads in each pooled dataset to specific sequenced samples based on the 5 bp barcodes. Illumina short reads were mapped to the reference sequence (including genomic sequence, chloroplast sequence and rRNA genes) using the Bowtie2 algorithm with no more than 2 mismatches (Langmead et al. 2009). Only the short reads with average base quality ≥20 (Phred quality score) and mapping quality ≥20 (Phred quality score) were considered for calling genetic variants. The length of the sequenced fragments was determined for pairs of mapped reads using the mapping coordinates of each mapped read.

### Genetic variant discovery and genotyping

The alignment results were processed using SAMtools mpileup to identify candidate genetic variants among all sequenced samples, including both SNPs and INDELs. To generate high quality and reliable genetic variants, several filtering criteria were applied to each candidate polymorphism locus: (1) mapping depth ≥10 reads in each sample; (2) genotype quality ≥20 (Phred quality score) in each sample; (3) no missing genotype information for each of the two parental lines; (4) at least 7 offspring samples with genotype information; (5) the identified alleles in offspring samples were consistent with the alleles in parental lines. For *Arabidopsis* samples, an additional filtering step was used to confirm the identified genetic variants. We downloaded the annotated genetic polymorphism data between Col and Ler ecotypes from the TAIR database (https://www.Arabidopsis.org/browse/Cereon/index.jsp), and only the candidate polymorphisms present in this dataset were used in further analysis. Potato polymorphisms with more than two alleles were excluded from further analysis.

### Sequence variant validation using Sanger sequencing

*Arabidopsis* ecotypes, Col and Ler, have been sequenced by Sanger sequencing and the genome wide polymorphisms between the two ecotypes have been annotated in TAIR database (https://www.Arabidopsis.org/browse/Cereon/index.jsp). We used these Sanger sequencing identified sequence variants as validation of those identified from the Illumina sequencing data for Arabidopsis data. The relevant polymorphism data is; however, not available for potato RAD-seq data. We randomly selected 50 identified polymorphisms identified from the potato RAD-seq sequence reads, 1/3 of which had a sequence high coverage (≥100), 1/3 had a medium coverage (50–100) and the rest had a low coverage (10–50), then re-sequenced them using Sanger sequencing and evaluated the genotype result among potato diploid and tetraploid parental lines.

### Genotype validation

To validate the genotypes inferred from the RAD-seq data, the genotype frequency (including two homozygous genotypes and the heterozygous genotype) of each polymorphic locus in offspring samples was estimated. The Pearson’s Chi-square test was implemented to test for the goodness of fit between observed and expected genotype frequency, with degrees of freedom equal to 2:

1 $${\text{Pearson's}}\,\;x^{2} = \sum\limits_{i = 1}^{3} {(O_{i} - E_{i} )^{2} /E_{i} }$$

where, *O*_*i*_ is the observed genotype frequency for the *i*th genotype and *E*_*i*_ is the expected frequency for the *i*th genotype. To account for multiple tests we set the significance threshold at *P* < 0.05 after the Sidak correction (Westfall and Young 1993).

## Results

### Prediction of the optimal combination of restriction enzymes (REs)

Design of an optimized RAD-seq experiment is realized by selecting a set of suitable RE(s) to cut the genome of interest, subject to a series of both general and specific optimization criteria. We focused here on plant genomes only. The optimizing criterion in general is to identify an adequate number of uniformly distributed genetic polymorphisms for genotyping a large population of plant species with known genome sequence at the lowest possible cost using next generation sequencing technologies such as the Illumina platform. Specifically, optimization was performed to maximize the number of genomic DNA sequence reads that uniformly cover the plant genome under study and minimize the number of the sequence reads representing chloroplast DNA and rRNA genes. These optimizations need to be achieved for a given genome size of the target species, the number of genomic regions required to be sequenced, the desired marker density and the coverage required for meeting the experimental objectives.

An optimized RAD-seq experimental design for a plant species was initiated with 4 widely used restriction enzymes (EcoRI, HindIII, MspI and MseI) and sequence information of the genome under study. The bioinformatic analysis developed in the present study searched over all combinations of these REs for their cutting sites in the target genome and hence for the optimal pair that generated the desired number of DNA fragments (here, 10,000) within the target size range (224-424 bp, an appropriate length for Illumina sequencing) and covering the target genome as uniformly as possible. There is no technical difficulty to incorporate more REs into the search whenever needed.

Among the DNA segments generated, those representing chloroplast DNA and rRNA genes are inevitable. Chloroplast and rRNA genes can make up a significant fraction of total cellular DNA due to their extremely high copy number (Bock 2007). For example, it is estimated that there are 700 ± 60 copies of rRNA genes per haploid genome and 1000–1500 copies of chloroplast DNA per cell in different developmental stages of *Arabidopsis* leaf tissue (ecotype Columbia) (Zoschke et al. 2007). High copy numbers of chloroplast DNA have also been observed in various other plant species (S2 Table). If these fragments are not removed prior to sequencing of a plant RAD-seq library then a substantially large proportion of sequencing reads could be produced from the chloroplast DNA or rRNA genes. We proposed here a novel strategy involving a second round of restriction enzyme digestion to remove chloroplast sequence and rRNA genes from the selected fragments (determined from the first round of digestion) prior to sequencing, as illustrated in Fig. 1. The bioinformatic analysis surveyed all 269 commercially available REs to seek a combination of REs that could digest all of the fragments from chloroplast sequence and rRNA genes while keeping as many of the targeted genomic fragments intact as possible. To ensure computational efficiency and data storage, we have represented the computer programs in Fortran 90 developed in the present study to optimize the RAD-seq design, which can be found at http://www.statisticalgenetics.info/software.html together with a step by step guide. Through the website, one can find out executable codes of the compiled FORTRAN programs and a detailed guide on how to install the supporting compiler software to run the programs on a Linux operating system.

### Optimized RAD-seq experiments with diploid and autotetraploid *Arabidopsis* and potato

We experimentally tested the optimization process proposed above by firstly sequencing 4 pooled libraries, each of which consisted of 12 DNA samples and represented one of 4 plant populations. Each of the four populations was made up of two parental varieties and their 10 offspring in diploid or autotetraploid *Arabidopsis* or potato *Solanum tuberosum* (see “Materials and methods” for details of these parental *Arabidopsis* and potato lines).

We proposed to sequence approximately 10,000 fragments within a length range of 224–424 bp for each of 48 samples. For *Arabidopsis*, this number was comparable with previously published RAD-seq experiments (Truong et al. 2012) and will enable an average coverage of 125× to be achieved in these targeted regions with collection of an expected 1.25 M short reads per sample in 12-sample pooled libraries. We expected to identify an average of at least 1 genetic polymorphism per cM and would check this using the publically available polymorphism information between *Arabidopsis* Columbia (Col) and Landsberg (Ler) ecotypes. For potato, we did not have any available genome-wide polymorphism information for the parental lines used to create the diploid and autotetraploid offspring populations. However, we expected to be able to collect a high density of markers due to the high level of heterozygosity of the potato genome (Xu et al. 2011). We therefore designed a RAD-seq experiment to sequence 10,000 fragments evenly covering the potato genome, which far exceeds the number of genomic regions obtained in the previous work that was based on a genome site capturing technique (Uitdewilligen et al. 2013). Given collection of 2 M short reads per sample from 12-sample pooled libraries, we could expect an average coverage of 200× to be achieved in the targeted regions, which was sufficient for potato genotyping.

For the *Arabidopsis* genome, the bioinformatic analysis showed that the combination of EcoRI and MseI, as recommended for *Arabidopsis* RAD-seq previously (Alonso-Blanco et al. 1998; Truong et al. 2012), yielded 11,080 targeted fragments and recovered 994 annotated genetic variants between Col and Ler ecotypes. However, these 11,080 targeted fragments included 46 fragments from chloroplast sequence and rRNA genes. We chose to further investigate use of a new combination of REs (EcoRI, HindIII and MspI) to cut the *Arabidopsis* genome, based on a predicted 20,789 targeted fragments (nearly double the required number), covering almost double (1910) the number of annotated polymorphisms between Col and Ler (Table 1a). For the potato genome, the combination of EcoRI and MspI was selected for further investigation as it yielded an expected 34,603 targeted fragments, with only 22 fragments from chloroplast sequence and rRNA genes (Table 1b). While other combinations such as HindIII/MspI could yield more fragments, this would reduce the average sequencing coverage that could be achieved for the given number of short reads to be collected per sample.

**Table 1:** The number of sheared DNA fragments based on different RE combinations

Restriction enzyme combinations	Total fragments (coverage)	Selected fragments* (coverage)	Detectable variants	Fragments in rRNA and chloroplast regions
(a) *Arabidopsis* genome				
EcoRI, MseI**	68,867 (9.24 %)	11,080 (2.8 %)	994	46
EcoRI, MspI	53,005 (37.5 %)	9145 (2.4 %)	814	38
HindIII, MseI	118,721 (14.4 %)	17,566 (4.5 %)	1592	15
HindIII, MspI	77,638 (44.9 %)	14,624 (3.9 %)	1371	16
EcoRI, MseI, MspI	69,680 (7.5 %)	9219 (2.3 %)	782	33
HindIII, MseI, MspI	121,112 (12.1 %)	14,903 (3.7 %)	1353	11
EcoRI, HindIII, MseI	176,921 (20.6 %)	25,314 (6.42 %)	2288	55
** EcoRI, HindIII, MspI**	**101,787 (47.7** **%)**	**20,789 (5.5** **%)**	**1910**	**44**

Restriction enzyme combination	Total fragments (coverage)	Selected fragments* (coverage)	Fragments in rRNA and chloroplast regions
(b) Potato genome			
** EcoRI, MspI**	**244,772 (46.5** **%)**	**34,603 (1.6** **%)**	**22**
HindIII, MspI	272,706 (47.7 %)	40,769 (1.8 %)	23
EcoRI, MseI	403,491 (8.9 %)	56,315 (2.4 %)	46
HindIII, MseI	473,702 (9.9 %)	62,140 (2.7 %)	12

The RE combinations recommended in the current study are shown in bold

* Size range from 224 to 424 bps

** Recommended in (Alonso-Blanco et al. 1998; Truong et al. 2012)

Of the *Arabidopsis* targeted fragments, 44 were from chloroplast sequence and rRNA genes. We estimated that these 44 chloroplast and rRNA fragments could occupy as high as 40–60 % of sequence reads in diploid or tetraploid *Arabidopsis* (Table 2). Based on the bioinformatic search, we proposed use of three REs (SnaBI, StuI and TfiI) to remove all 44 chloroplast and rRNA fragments (Table 3a), leaving 8,025/20,789 (40 %) targeted genomic fragments (totaling 2.01 Mb) intact for RAD-seq library sequencing. This design met our target for sequencing approximately 10,000 fragments and for identifying polymorphisms at an average density of at least 1 marker per cM (953 annotated polymorphisms in a genome of size of 451 cM). For potato, two REs (HhaI and HinfI) were chosen from the bioinformatic prediction, and this combination of REs could remove all 22 chloroplast and rRNA fragments, and leave 11,018/34,603 (32 %) targeted genomic fragments (totaling 2.00 Mb) intact (Table 3b). The DNA fragments after this round of RE digestion were randomly and evenly distributed across the whole genome of *Arabidopsis* or potato (S1 Figure), confirming the suitability of the chosen REs for genome-wide RAD-seq in both species.

**Table 2:** Predicted proportions of sequence reads to be generated from the 20,789 selected DNA fragments

	Diploid *Arabidopsis*			Tetraploid *Arabidopsis*		
	Genome	rRNA	Chloroplast	Genome	rRNA	Chloroplast
Number of selected DNA fragments per haploid genome	20,745	4	40	20,745	4	40
Number of copies	2	2 × 700	1200	4	4 × 700	1200
Number of selected DNA fragments per cell	41,490	5600	48,000	82,980	11,200	48,000
Total number of selected DNA fragments per cell	95,090			142,180		
Proportion of reads mapped to different regions	**43.6** **%**	5.9 %	50.5 %	**58.4 %**	7.9 %	33.7 %

**Table 3:** The optimized combination of REs and their cut sites in each of three types of *Arabidopsis* and potato DNA fragments

RE combination	Cut sites within *Arabidopsis* fragments			Genomic DNA fragments intact	Annotated polymorphisms
	rRNA Fragments	Chloroplast fragments	Genomic DNA fragments		
(a)					
**SnaBI, StuI, TfiI**	4	40	12,720	8025	953
AvaII, TfiI	4	40	13,669	7070	817
Sse9I	4	40	17,488	3215	376

RE combination	Cut sites within potato fragments			Genomic DNA fragments intact
	rRNA Fragments	Chloroplast fragments	Genomic DNA fragments	
(b)				
**HhaI, HinfI**	2	20	23,563	11,018
CviJI	2	19	31,298	3284
BfuCI	2	17	21,672	12,912

### Illumina sequencing of the pooled RAD-seq libraries

**Fig. 2:**
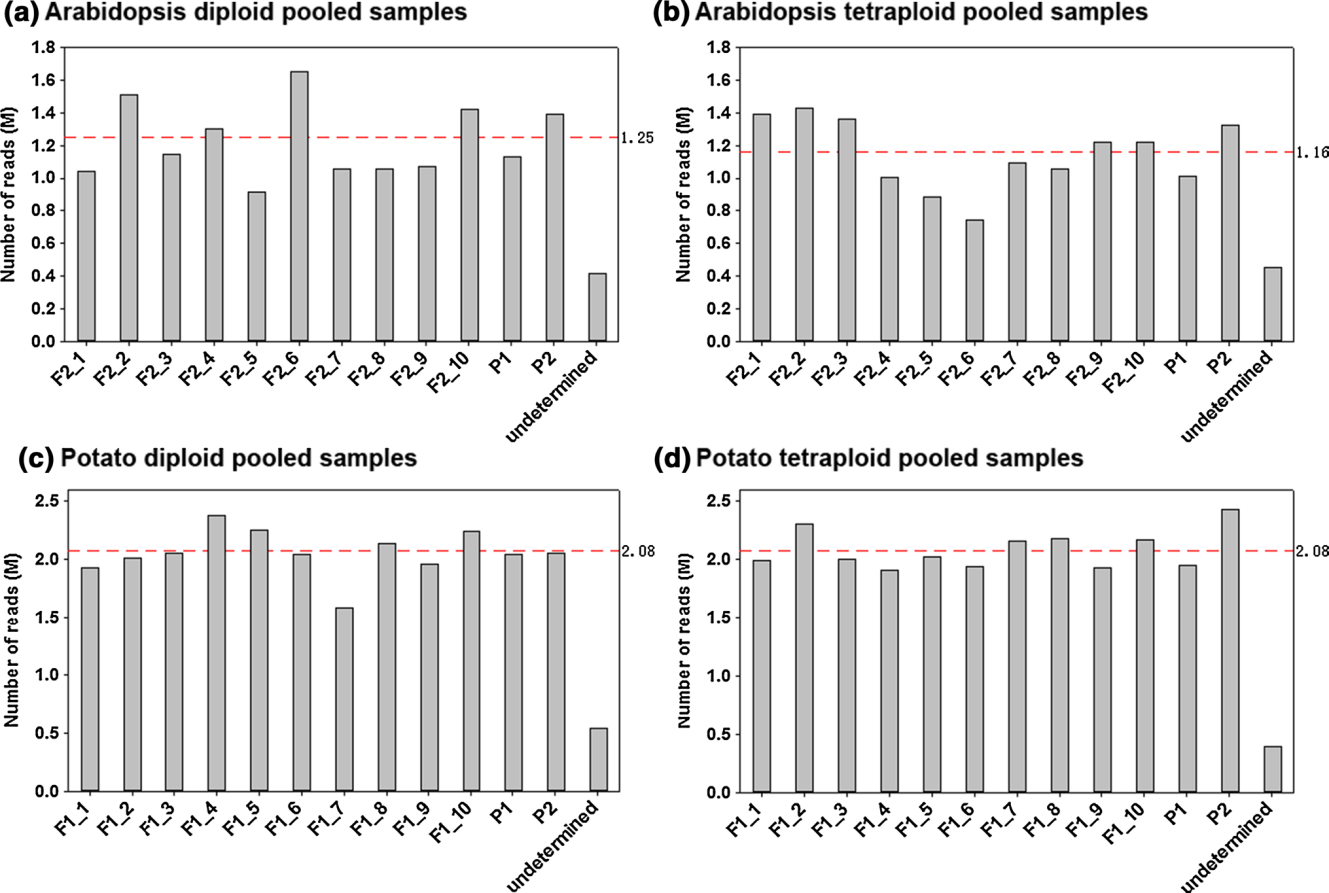
Distribution of the number of short reads across 12 *barcoded* samples in each pooled RAD-seq dataset. The *red dashed line* shows the average number of paired reads per sample

We prepared and sequenced a total of 6 pooled libraries (Table 4). The first 4 libraries underwent two rounds of RE digestion using the selected enzymes from the above analyses to cut the genomic DNA into required segments and then remove the DNA fragments from chloroplast and rRNA genes. Approximately 15 and 24 M short reads were collected from sequencing each of the *Arabidopsis* and potato pooled libraries respectively. The distribution of the number of short reads obtained for each sample in the pooled sequence libraries was shown in Fig. 2 and S3 Table. For every 12-sample pooled dataset, the expected proportion of reads per sample was 8.33 %, corresponding to 1.25 M short sequence reads per sample in *Arabidopsis* and 2.00 M reads per sample in potato (Table 4). In each pooled dataset, the proportion of reads from each sample ranged from 7.00 to 10.00 %. No more than 3 % of reads in each pooled dataset were unassigned to a sample, suggesting a good control of sequencing or PCR errors. The data shows an even distribution of reads across the pooled samples (one sample student *t*-test, *P* value ≥0.10; the coefficient of variation = 8.0–19.4 %). To evaluate the efficiency of removing chloroplast and rRNA DNA fragments, we constructed a further 2 control RAD-seq libraries by conducting only the first round of RE digestion (Table 4). Approximately 3 and 6 M reads were produced for the *Arabidopsis* and potato libraries, respectively and these were evenly allocated to samples according to the barcodes (one sample student *t* test, *P* value >0.10) (S4 Table).

**Table 4:** Summary of 6 pooled DNA sequencing libraries constructed using the optimized RAD-seq design (the first four) and the corresponding control design (the last two) and the number of sequence reads expected from each of the pooled sequence libraries

	*Arabidopsis*		Potato		*Arabidopsis* (control)	Potato (control)
	Diploid	Tetraploid	Diploid	Tetraploid		
Sequenced samples	2 parents + 10 offspring	2 parents + 10 offspring	2 parents + 10 offspring	2 parents + 10 offspring	2 parental lines + 1 offspring (diploid and tetraploid)	2 parental lines + 1 offspring (diploid and tetraploid)
1st digestion	EcoRI, HindIII, MspI	EcoRI, HindIII, MspI	EcoRI, MspI	EcoRI, MspI	EcoRI, HindIII, MspI	EcoRI, MspI
2nd digestion	SnaBI, StuI, TfiI	SnaBI, StuI, TfiI	HhaI, HinfI	HhaI, HinfI	–	–
Sequencing platforms	Illumina MiSeq	Illumina MiSeq	Illumina HiSeq 2000	Illumina HiSeq 2000	Illumina MiSeq	Illumina HiSeq 2000
Sequencing lengths (bp)	2 × 150	2 × 150	2 × 100	2 × 100	2 × 150	2 × 100
Expected coverage	125	125	200	200	60	100
Expected number of reads per sample	1.25 M	1.25 M	2.00 M	2.00 M	0.50 M	1.00 M
Total number of reads	15 M	15 M	24 M	24 M	3 M	6 M

### The efficiency to remove the chloroplast and rRNA DNA fragments

Raw short reads were aligned to either the *Arabidopsis* or potato reference sequence (including genomic, chloroplast and rRNA gene sequences) using Bowtie2 (Langmead et al. 2009). Over 85 % of short reads were successfully mapped to the reference with no more than 2 mismatches per read (Table 5), excluding the possibility of contamination during library preparation and sequencing. In the *Arabidopsis* datasets, only around half of all reads could be aligned to genomic sequence in diploid (47 %) and tetraploid (53 %) samples when chloroplast and rRNA sequences were not removed during library construction, while a substantial 43 % (in diploids) and 39 % (in tetraploids) were mapped to chloroplast and rRNA sequences (Table 5). Similarly, less than one-third of potato RAD-seq reads could be aligned to genomic sequence in diploid (27 %) and tetraploid (30 %) potato samples, while the majority of reads mapped to chloroplast and rRNA sequences (Table 5). These results were consistent with our bioinformatic prediction based on the extremely high copy number of chloroplast and rRNA gene DNA sequences in plant cells (Table 2). In contrast, when chloroplast and rRNA sequences were removed using a second round of digestion, the majority of reads were successfully mapped to genomic sequence (Table 5), both in *Arabidopsis* diploids (82 %) or tetraploids (81 %), and in potato diploids (83 %) or tetraploids (83 %). Only a small minority of *Arabidopsis* or potato reads (3–10 %) mapped to chloroplast sequence and rRNA genes (Table 5). These observations indicate the objectives of the optimized RAD-seq approach developed in the present study have been successfully achieved to effectively minimize presentation of the chloroplast and rRNA genes in the sequence libraries and to significantly increase the proportion of reads mapped to genomic sequence.

**Table 5:** Percentage of RAD-seq short reads aligning to different genome regions in *Arabidopsis* and potato

	*Arabidopsis*				Potato			
	Chloroplast and rRNA fragments unremoved		Chloroplast and rRNA fragments removed		Chloroplast and rRNA fragments unremoved		Chloroplast and rRNA fragments removed	
	Diploid	Tetraploid	Diploid	Tetraploid	Diploid	Tetraploid	Diploid	Tetraploid
Genomic	**47.4**	**53.2**	**81.9**	**81.6**	**27.0**	**30.3**	**83.4**	**82.9**
Chloroplast	31.4	27.0	6.1	6.1	64.5	61.1	5.5	2.7
rRNAs	11.9	11.6	3.0	3.1	0.7	1.2	0.1	0.1
Unmapped	9.3	8.2	9.0	8.7	7.8	7.4	11.0	14.4

### Coverage and consistency of targeted fragments from the optimized RAD-seq protocol

**Fig. 3:**
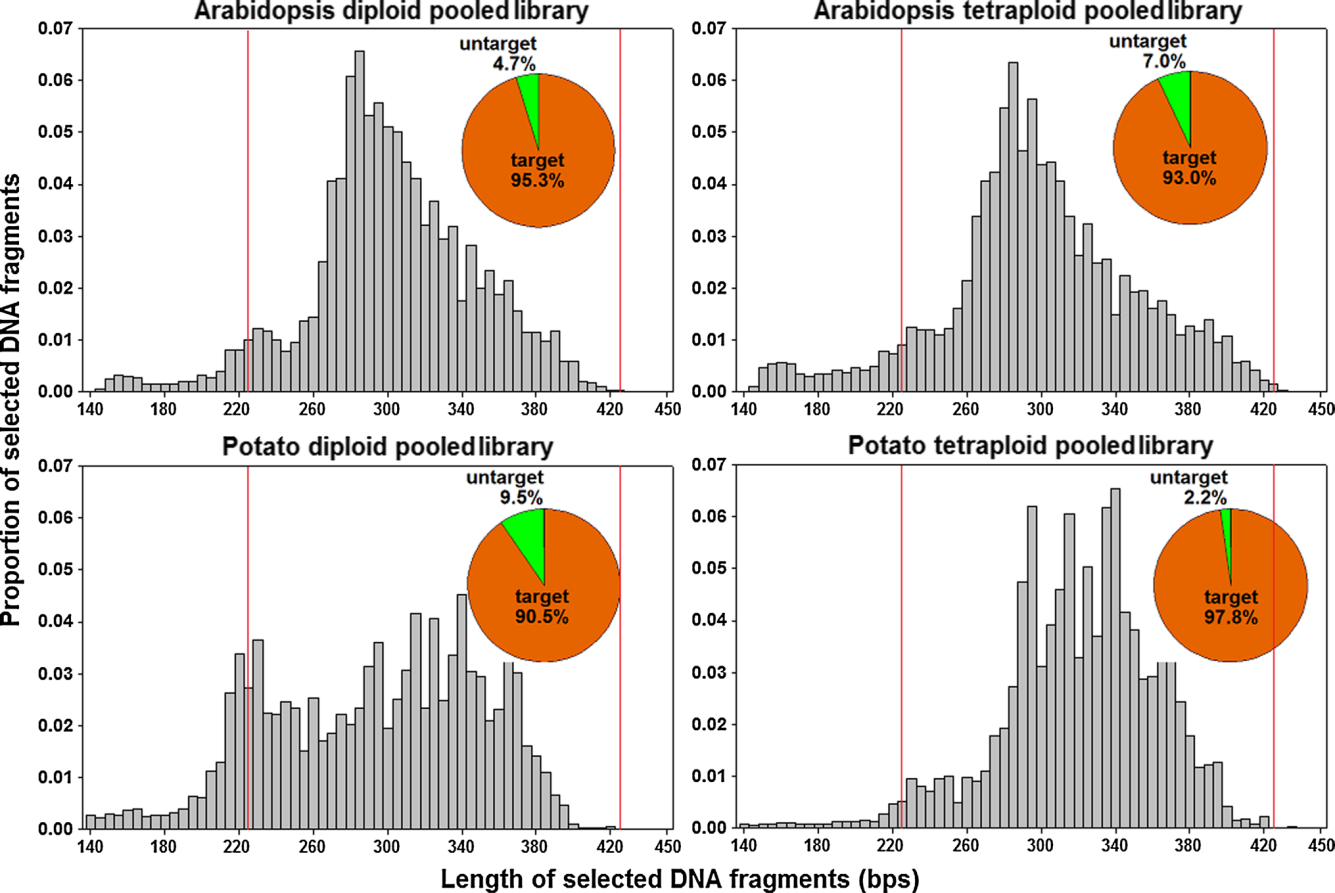
Length distribution of sequenced DNA fragments in each pooled RAD-seq dataset

Since RAD-seq is a type of reduced-representation genomic sequencing approach, it is crucial for genotyping purposes that the same genomic fragments are selected among different pooled samples. To achieve this, we used a double size-selection strategy via the Pippin Prep automated size selection technology to further optimize the RAD-seq experimental design proposed in the present study (see S1 Protocol). To evaluate its performance, we firstly analyzed the proportion of sequenced fragments falling within the selected size range (from 224 to 424 bp). The length distribution of sequenced DNA fragments for each pooled RAD-seq dataset was shown in Fig. 3, which clearly demonstrated that more than 90 % of sequenced fragments in each pooled RAD-seq dataset fell within the target size range.

We next analyzed the coverage of sequence reads across the whole genome and in the selected genomic regions for each sequenced sample, and the corresponding consistency between deeply mapped regions among pooled samples. In the *Arabidopsis* RAD-seq datasets, there were on average 1.25 M 2 × 150 bp paired-end reads generated for each sample (Fig. 2). About 4.96-8.68 Mbp genome-wide genomic regions were covered by more than 2 reads in each diploid and tetraploid sequenced sample; meanwhile, there were about 2.13–3.18 Mbp genomic regions deeply covered by at least 10 reads in each sample (Table 6). In these deeply covered regions, about 1.87 Mbp and 1.92 Mbp genomic regions were consistently covered in at least 10 of the 12 diploid and tetraploid samples, respectively. Among the bioinformatically selected 2.01 Mbp genomic regions consisting of 8,025 small genomic fragments, about 1.54-1.93 Mbp (76.6–96.0 %) were uniquely covered by at least 2 reads in each diploid and tetraploid sequenced sample. Deep coverage of at least 10 reads was achieved for 1.42–1.83 Mbp (70.6–91.0 %) selected regions in each sequenced sample (Table 6). Importantly, 1.41 and 1.42 Mbp (70.1 and 70.6 %) of selected regions were consistently and deeply covered among at least 10 of the 12 diploid and tetraploid sequenced samples, respectively. The optimized potato RAD-seq data showed a similar performance. There were on average 2 M 2 × 100 bp paired-end reads generated for each sample (Fig. 2). Only 4.11-5.61 Mbp genomic regions were covered and about 2.16–3.02 Mbp regions were deeply mapped in each sequenced diploid and tetraploid sample (S5 Table). Among the deeply covered regions, 1.71 and 1.59 Mbp regions were consistently covered across at least 10 of the 12 diploid and tetraploid samples, respectively. In the bioinformatically selected 2.0 Mbp regions consisting of 11,018 genomic fragments, about 1.45–1.60 Mbp (72.5–80.0 %) regions were uniquely covered by at least 2 reads in each diploid and tetraploid sequenced sample, respectively, while deep coverage was achieved for 1.27–1.52 Mbp (63.5–76 %) regions in each sequenced sample (S5 Table). Importantly, 1.20 and 1.13 Mbp (60 and 56.5 %) of bioinformatically selected regions were consistently deeply covered in at least 10 of the 12 diploid and tetraploid sequenced samples, respectively. These results demonstrate that the optimized RAD-seq approach is robust in selecting the targeted genomic fragments for deep sequencing in a consistent way across different samples in the pool.

**Table 6:** Coverage of *Arabidopsis* RAD-seq reads in Mbp across the whole genome and in selected regions (2.0 Mbps)

Sample ID	Diploid sample pools				Tetraploid sample pools			
	Whole genome		Selected regions		Whole genome		Selected regions	
	Covered*	Deep**	Covered*	Deep**	Covered*	Deep**	Covered*	Deep**
F2_1	6.95	2.73	1.86	1.69	5.74	2.88	1.89	1.76
F2_2	8.51	3.17	1.93	1.83	8.38	3.08	1.91	1.80
F2_3	7.36	2.75	1.83	1.69	5.50	2.88	1.84	1.68
F2_4	7.69	2.81	1.76	1.68	6.97	2.60	1.88	1.63
F2_5	4.96	2.23	1.68	1.60	7.76	2.39	1.87	1.55
F2_6	5.97	3.18	1.84	1.74	5.94	2.13	1.84	1.70
F2_7	5.55	2.55	1.82	1.63	7.60	2.68	1.82	1.62
F2_8	8.68	2.61	1.82	1.61	5.10	2.53	1.84	1.60
F2_9	7.41	2.64	1.81	1.66	8.28	2.87	1.90	1.72
F2_10	5.79	3.00	1.81	1.69	5.69	2.76	1.88	1.69
P1	6.05	2.54	1.87	1.53	5.34	2.23	1.83	1.69
P2	5.57	2.81	1.57	1.46	5.63	2.86	1.54	1.42

* At least 2 reads uniquely mapped

** At least 10 reads uniquely mapped

### Sequence variant and genotype calling

For both *Arabidopsis* and potato RAD-seq datasets, variants were called from sequence data of both parental lines and their segregating offspring. Only uniquely mapped reads with mean base quality ≥20 (Phred score) and mapping quality ≥20 (Phred score) were used for detecting candidate genetic polymorphisms using the SAMtools mpileup algorithm (Li et al. 2009). Various genetic variants detected from the RAD-seq datasets were summarized in Table 7. The number of genetic variants detected in potato samples was 3–4 times larger than the number detected in *Arabidopsis*. Given the similar depth of coverage of 2–3 Mbp genomic regions across both *Arabidopsis* and potato genomes, this result likely reflects the higher density of genetic polymorphisms in the potato populations. Only less than 1 % of potato genome polymorphisms were tri-or tetra- allelic and these were excluded from further analysis.

**Table 7:** The number of genetic variants detected from the optimized Arabidopsis and potato RAD-seq datasets

	Arabidopsis		Potato	
	Diploid	Tetraploid	Diploid*	Tetraploid*
Candidate variants	39,313	40,076	174,447 (1058)	125,291 (1087)
SNPs	28,779	28,649	158,272 (679)	114,407 (797)
INDELs	10,534	11,427	16,175 (379)	10,885 (290)
Verified variants	848	786	1779	5045
SNPs	601	554	1673	4688
INDELs	247	232	106	357

* The numbers in parentheses refer to tri- and tetra- allelic genetic markers

**Fig. 4:**
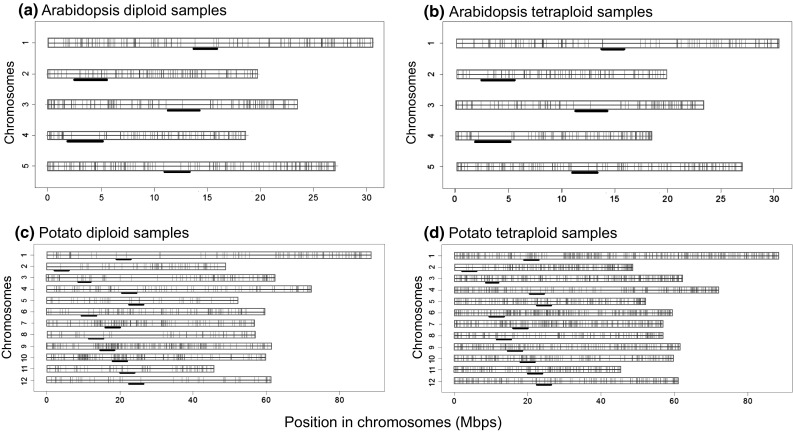
Distribution of detectable genetic markers in the *Arabidopsis* and potato genomes. The *black bars below* each chromosome indicate the centromere regions

Although a large number of polymorphisms were identified from the sequence data, we implemented stringent filtering criteria to derive high quality genetic variants as detailed in Materials and Methods. In the *Arabidopsis* data, 848 and 786 high quality genetic polymorphic sites passed the filtering criteria in diploid and tetraploid samples, respectively (Table 7). In the potato RAD-seq data, 1779 and 5045 such high quality polymorphic markers were identified in the diploid and tetraploid samples, respectively. The distribution of high quality genetic markers across the whole genome was shown in Fig. 4, which illustrates that the genetic markers finally selected are dispersed evenly across the whole genome, excluding the centromere regions. For *Arabidopsis*, we divided the 120 Mbp genome into 450 approximately equal (1 cM) intervals. There were 311 (69 %) and 302 (67 %) intervals with at least 1 detected polymorphism in diploid and tetraploid *Arabidopsis* samples, respectively. For potato, the 725 Mbp genome was equally divided 725 intervals of 1 Mbp. There were 453 (62 %) and 633 (87 %) intervals with at least 1 detected genetic polymorphism in diploid and tetraploid samples, respectively. Furthermore, the distribution of genetic variants detected from the present potato RAD-seq data covered the whole genome more uniformly when compared with previously published genotyping-by-sequencing analysis using sequence capture (S2 Figure) (Uitdewilligen et al. 2013). It was clear that, as expected, the regions surrounding the centromeres were usually covered by fewer polymorphic markers in both *Arabidopsis* and potato genomes.

The base quality (BQ) and mapping quality (MQ) implemented here are comparable to those in Clevenger et al. (2015) who set MQ ≥30 and BQ ≥20 for screening reads from a polyploid sequencing experiment and in Uitdewilligen et al. (2013) who used MQ ≥13 and BQ ≥13 in an autotetraploid potato sequencing experiment. We compared the rate of sequence reads screened using BQ ≥30, 20 and 13 and the rate of mapping of sequence reads using MQ ≥30, 20 and 13 from the sequence datasets from the diploid and autotetraploid Arabidopsis and potato experiments. The analysis showed that 95–96 % of the sequenced nucleotide bases called at BQ ≥13 were also called at BQ ≥30 as implemented in the present study. Moreover, there was only a 0.6–3 % increase in mapped sequence reads across our diploid and tetraploid Arabidopsis and potato sequence datasets when MQ decreased from ≥30 to ≥13. These indicate that use of more or less stringent BQ and MQ than that in the present study does not lead to marked difference in the calling and mapping rates of the sequence reads, at least in the current sequence datasets. Up to 98 % of the polymorphic markers identified in the diploid and autotetraploid potato samples were common between those screened at these two levels of quality control.

To confirm the above genotyping analysis and further examine the accuracy of the SNP and small INDEL calls, the annotated polymorphism information between Col and Ler ecotypes was downloaded from the TAIR database and used to validate our genotype calls made among the Arabidopsis Col and Ler parental ecotypes. Among the 940 and 965 annotated polymorphic positions genotyped using the RAD-seq data from diploid and tetraploid *Arabidopsis* parental lines, 98.1 and 97.4 % of the polymorphism calls were consistent with the ‘gold standard’ information from the annotated polymorphism database. The annotated polymorphism information was not available for the diploid and autotetraploid potato parents. We randomly selected 50 polymorphisms called from the RAD-seq experiments for the diploid and autotetraploid potato parental varieties, and compared with those identified from Sanger sequencing. To account for variation in sequence coverage, we selected the 50 tested polymorphisms with 1/3 with high coverage in the RAD-seq data (≥100), 1/3 with medium coverage (50–100) and 1/3 with low coverage (10 ~ 50). The consistency between RAD-seq genotyping and Sanger sequencing reached 97.0 and 96.0 % for diploid and autotetraploid potato varieties, respectively.

### Validating the genotyping results using marker segregation analysis

**Fig. 5:**
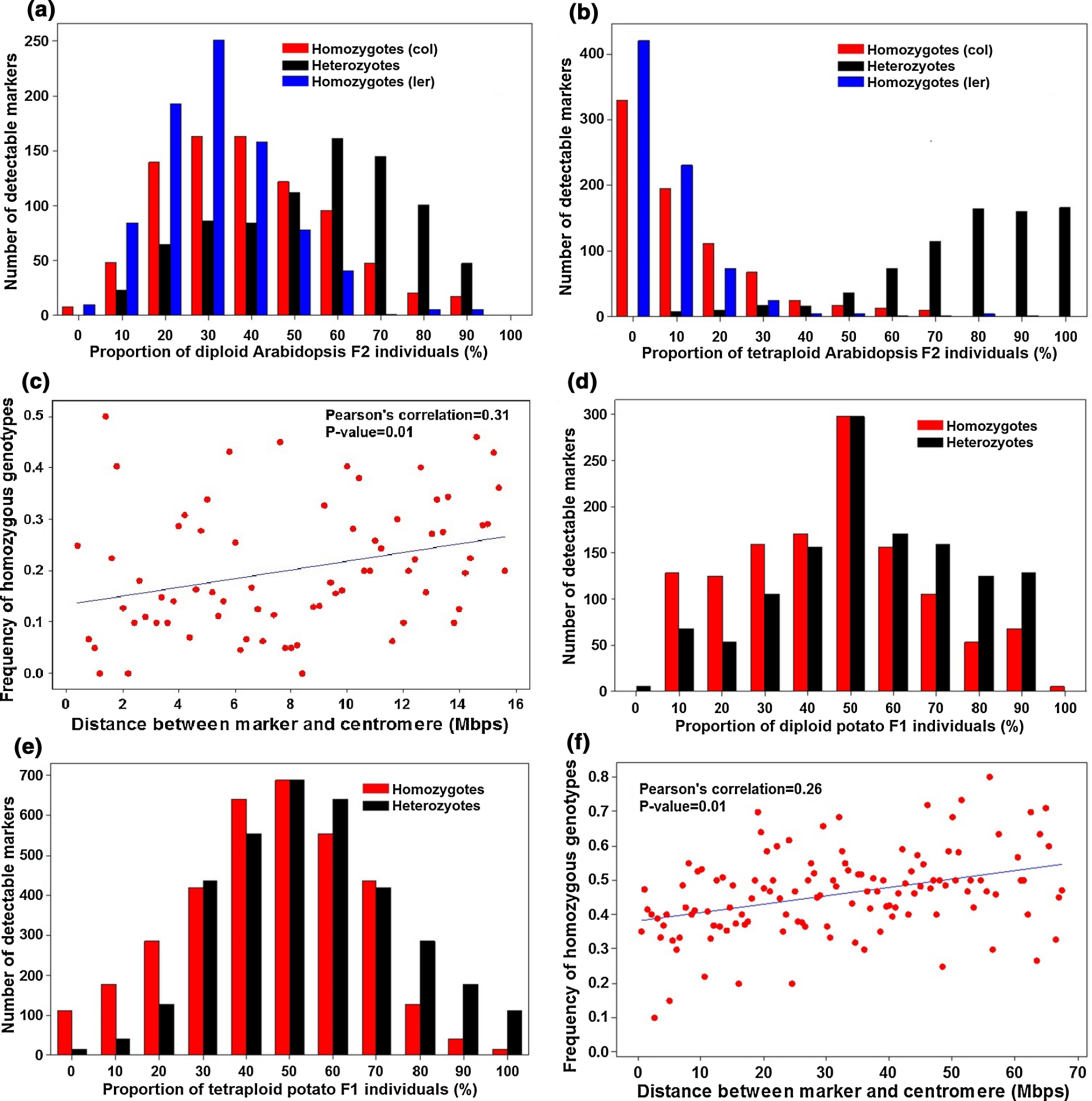
Distribution of genotype frequency in offspring samples. Distribution of three possible genotypes (homozygote, heterozygote and homozygote) at 846 and 786 detected SNP sites from the diploid *Arabidopsis* F2 samples (**a**) and in tetraploid *Arabidopsis* F2 samples (**b**) respectively. The correlation between marker homozygous genotype frequency and distance to the centromere region in *Arabidopsis* tetraploid F2 samples (**c**). Distribution of three possible genotypes (homozygote, heterozygote and homozygote) at 1269 and 3499 detected SNP sites from the diploid potato F1 samples (**d**) and in tetraploid potato F1 samples (**e**). The correlation between marker homozygous genotype frequency and distance to the centromere region in potato tetraploid F1 samples (**f**)

For both diploid and tetraploid *Arabidopsis* samples, we estimated the genotype frequencies for each marker across all sequenced segregating offspring samples (Fig. 5a, b). In *Arabidopsis* diploid F_2_ offspring, the average observed genotype frequencies of 846 markers were 30 %: 48 %: 22 % (homozygous genotype with Col: heterozygous: homozygous genotype with Ler), which was very close to the expected frequencies (25 %: 50 %: 25 %). We implemented the Chi-square goodness of fit test for each of the 848 detected markers and found none of the markers significantly deviated from the expected frequencies at a *P* value of 0.05 (after Sidak correction). It should be noted that the expected frequencies of genotypes at a biallelic marker were 6 %: 88 %: 6 % in the *Arabidopsis* tetraploid F2 samples when double reduction was set to be absent (Luo et al. 2000). The average observed genotype frequencies among the markers were 12 %: 80 %: 8 %. Of 786 genetic markers, 133 showed significant deviation from the expected frequencies (Chi-square test, *P* value <0.05, after Sidak correction), with the homozygous genotypes showing significantly higher frequencies than expected in each case. We found that the frequencies of homozygous genotypes at those significant markers increased as the distance between the marker and the centromere increased (Pearson correlation coefficient = 0.31, *P* value = 0.01, Fig. 5c). This suggests that the observed overrepresentation of homozygous genotypes can likely be explained by double reduction at these marker loci (Luo et al. 2000). We would like to acknowledge here that the term ‘genotype’ in tetraploid here is not in its strict sense because it contains only information of constituent alleles but no information of the allelic dosage. On the other, characterization of tetraploid genotype distribution in a segregating autotetraploid population such as F_2_ needs consideration of the double reduction parameter as described elsewhere (Luo et al. 2000). Given a small sample size of 10 offspring, we will not exploit this in any further here.

Given the high degree of heterozygosity of potato parental lines and for demonstration purposes, we considered here only those SNP markers at which one parental line was homozygous (e.g., AA) and the other parental line was heterozygous (e.g., Aa). In the potato diploid offspring population, the expected genotype frequencies should be 50 %: 50 %: 0 % (homozygous ‘A’ genotype: heterozygous Aa genotype: homozygous ‘a’ genotype). The observed diploid genotype frequencies were 45 %: 55 %: 0 % (Fig. 5d). None of the 1269 identified markers was detected to deviate significantly from the expected genotype frequencies at a *P* value of 0.05 after Sidak correction. For a potato tetraploid offspring population, the expected genotype frequency distribution was expressed in term of α, the coefficient of double reduction, and summarized in S6 Table. The observed genotype frequencies were 46 %: 54 %: 0 % (Fig. 5e). Given the limitation of the offspring sample size, we did not fit the observed data to the model. Instead, we explored the distribution of homozygous genotype frequency of each marker with respect to the distance between the marker and the centromere region (Fig. 5f). Similarly to the *Arabidopsis* tetraploid samples, the homozygous genotype frequencies were significantly positively correlated with the distance between the marker and the centromere region (Pearson correlation coefficient = 0.26, *P* value = 0.01), indicating the likely occurrence of double reduction during meiosis.

## Discussion

Efficiently identifying and genotyping genome-wide polymorphisms in large populations lie at the heart of modern genomics and plays an essential role in tackling many fundamental questions in genetics, ecology and evolution. The RAD-seq (restriction site associated DNA sequencing) approach combines genotyping-by-sequencing with a reduced-representation strategy to make this goal considerably more time and cost-effective in comparison to conventional platforms such as microarray based genotyping technologies and whole genome DNA sequencing. Microarray and genomic DNA sequencing platforms typically cost hundreds to thousands of US dollars per sample, depending on the genome size. Meanwhile, the RAD-seq strategy reduces the cost to $20–$50 per sample (Elshire et al. 2011; Peterson et al. 2012), enabling sufficient mapping depth in selected genomic regions to be obtained with an acceptable cost for genotyping large populations in several animal (Baird et al. 2008; Gonen et al. 2014; Etter et al. 2011; Peterson et al. 2012; Wang et al. 2013) and plant (Chen et al. 2013; Chutimanitsakun et al. 2011; Elshire et al. 2011; Guo et al. 2014; Peterson et al. 2012; Pfender et al. 2011; Poland et al. 2012b; Romero-Severson et al. 2014; Truong et al. 2012; Wang et al. 2012a, b) species.

The current design of existing RAD-seq has a significant drawback which may greatly hinder its widespread and efficient use in plant species, particularly because DNA samples in almost all sequencing based genotyping studies in plant/crop species are prepared from leaf tissue (Chen et al. 2013; Chutimanitsakun et al. 2011; Elshire et al. 2011; Guo et al. 2014; Peterson et al. 2012; Pfender et al. 2011; Poland et al. 2012b; Romero-Severson et al. 2014; Truong et al. 2012; Uitdewilligen et al. 2013; Wang et al. 2012a, b, 2013) to ensure a high quality (integrity and quantity) of DNA available for sequencing. The current approaches have not addressed the large copy number (1000–10,000) of chloroplast sequence and rRNA genes (Chen et al. 2013; Romero-Severson et al. 2014; Truong et al. 2012; Uitdewilligen et al. 2013) in plant cells. These chloroplast DNA and/or rRNA genes may occupy a substantially large proportion (30–60 %) of sequence reads collected from standard RAD-seq protocols, and in turn, will significantly dilute coverage of the sequence reads across the genome under question. For example, Wang et al. recently conducted a RAD-seq experiment using the restriction enzyme MseI to genotype 100 grape DNA samples (Wang et al. 2012a). As expected, without proper removal of rRNA and chloroplast DNA from the leaf samples in the sequencing library construction, the experiment obtained the only a low proportion (37 M/117 M or 31.6 %) of sequence reads from all 100 sequenced samples, which could be used in further analyses (Wang et al. 2012a). This contrasts with the high proportion (at least 81 %) of reads mapped to genomic sequence using our optimized protocol in either diploid or tetraploid *Arabidopsis* or potato (Table 5). The authors suggested that the highly redundant sequence reads would be mapped to repeated regions of the grape genome. We carried out an in silico analysis to generate the DNA segments by using MseI to shear the grape genome (http://www.genoscope.cns.fr/externe/GenomeBrowser/Vitis/) and then aligned the DNA segments varying in length from 300 to 400 bp onto the reference genome (S7 Table). We found that, among the 142,722 DNA segments, 4 were from rRNA and 63 from chloroplast DNA, and that 95.7 % of the segments are not in fact aligned to the repeated regions. Thus, the highly redundant sequence reads must be attributable to the rRNA and chloroplast DNA sequences, indicating the importance of their removal during sequence library construction.

Wang et al. (2012b) proposed 2b-RAD, a type IIB restriction enzyme based approach for simplified genomic genotyping, and tested it with profiling genetic variants in a large population of *Arabidopsis thaliana*. Marshall and Halford thoroughly reviewed all available type IIB restriction enzymes (REs) and found none of the REs of this type may produce DNA segments longer than 40 bps (Marshall and Halford 2010). The short length of DNA segments generated by the IIB REs may significantly limit the mapping efficiency of the sequence reads produced. Given that current sequencing techniques such as Illumina can generate 2 × 300 bp sequence reads, the utility of the 2b-RAD approach may be greatly hindered. Secondly, the 2b-RAD method, unlike the method proposed here, was not designed to control the high proportion of non-genomic sequence reads from chloroplast DNA and rRNA genes. We re-analyzed the 2b-RAD sequence data by Wang et al. (2012b) and summarized the analyses in S8 Tables. It can be seen from the tables that a substantially large proportion of short sequence reads generated from the 2b-RAD method were predicted to come from the chloroplast DNA or rRNA genes (S8 Table). As expected, a substantial proportion of sequence reads (40–61 %) were not mapped to the reference genome, in sharp contrast to only about 10 % of unmapped sequence reads in the present optimized RAD seq experiments (Table 5, Table S8). Among the mapped short sequence reads achieved by the 2b-RAD method, approximately 30–39 % were mapped to non-genomic regions including rRNA genes or chloroplast DNA. These proportions were controlled to only 3–10 % in the present optimized RAD-seq experiments (Table 5).

In this paper, we present a new and optimized RAD-seq design, which integrates bioinformatic analysis and a novel library construction protocol, to maximize uniformity and coverage of sequence reads across the genome and to minimize presentation of chloroplast and rRNA DNA in the sequence reads for a given series of design parameters. Briefly, the optimization goal is achieved through use of an optimal combination of restriction enzymes (REs) in the construction of sequencing libraries using two rounds of RE digestion. The first round of digestion shears the genomic DNA into fragments in the length range required by a given sequencing platform, for example 224–424 bp for the Illumina sequencing technique. The second round of digestion further digests those fragments representing chloroplast sequence and rRNA genes. There are as many as 269 commercially available REs. The bioinformatic tools developed in the present study search various combinations of these REs using the information of their recognition cutting sites and the genome sequence under study, to find an optimal combination of REs that can be used in two rounds of restriction digestion to achieve the experimental objectives. Another important and distinct property of the optimized RAD-seq method is its in silico guided selection of DNA segments to be sequenced through use of combination of appropriate restriction enzymes. The bioinformatic tools and experimental protocols developed in the present study are generic for end-users to design and conduct the genome-wide identification and genotyping of genetic variants in large populations of plant or crop species according to the size of the genome of interest, required sequence coverage, amount of sequencing data required to be generated, the number and density of polymorphic sites to be targeted, and the designated genotyping budget. We would stress that the computational tools developed here are delivered in executable Fortran 90 programs rather than in more popular computer language such as R scripts though there is no technical difficulty to do so. This is entirely due to consideration of computational efficiency and limitation in memorizing sequence data of, a working genome and intermediate data generated in course of the analysis, in particular the genome has a large size like potato and the analysis involves a large number of samples. On the other hand, primary analyses of sequence data from the next generation sequencing technique like Illumina are usually conducted using a Linux supported computer cluster. Thus, we proposed end users to run the Fortran programs under a Linux operating system though the programs can also be run under the Windows OS. Nevertheless, these will not create any major difficulties even for an end user without specialist computer experience to implement and run the programs because the Fortran compiler is freely available to public and a detailed step by step guide for downloading the compiler and running the programs can be found from http://www.statisticalgenetics.info/software.html.

We experimentally tested the optimization strategy by implementing it to construct 4 pooled RAD-seq libraries, each of which is made up of 12 DNA samples and represents a population consisting of diploid or tetraploid parental lines and their 10 offspring plants of the model plant *Arabidopsis* or the crop plant potato (*Solanum tuberosum*). The pooled libraries were sequenced by two Illumina sequencers, MiSeq (*Arabidopsis*) and HiSeq 2000 (potato). By comparing the sequencing reads obtained from these optimized RAD-seq libraries to those obtained from control RAD-seq libraries, we demonstrated that 30–60 % of sequence reads are mapped to chloroplast sequences or rRNA genes if the DNA fragments representing these undesirable sequences (although in small number (20–40) are not properly removed during library construction. In the optimized RAD-seq experiments, these undesirable sequence reads can be brought down to only 3–10 %. Accordingly, the genome derived reads may be enriched from 27–53 % to 82–84 %, which are largely concentrated on the pre-designed locations. Use of the Pippin-Prep automated size selection in the optimized approach may control contamination of DNA segments of a length <244 bp to a level as low as 2.2–9.5 %. Thus, the optimized RAD-seq approach presented here effectively overcomes this potential hurdle and significantly increases the efficiency of RAD-seq to allow its widespread application to plant or crop species.

The optimized RAD-seq design confers flexibility in the selection of genome regions to be sequenced and thus enables uniform coverage of sequence reads to be generated across the genome under question. This is a practically important feature for implementing the approach for DNA molecular marker genotyping in large plant/crop populations for genetic linkage analyses or population genetic studies. With this feature, one can design the number, density and genome location of genetic markers to be targeted. In fact, mapping resolution depends on the effective number of recombinations, and thus use of an exceptionally dense set of markers does not necessarily lead to extra gain in improvement of gene mapping efficiency because the efficiency is largely determined by the number of recombinants in the mapping population (Mackay 2001). In this study, we planned to deeply sequence approximately 10,000 small regions uniformly covering the whole genome of *Arabidopsis* and potato. The results from the sequencing experiments showed that with an average of 1.25 M 2 × 150 bp paired-end reads generated for each diploid or tetraploid *Arabidopsis* sample, 1.54–1.93 Mbp (76.6–96.0 %) of selected genomic regions were covered by at least 2 reads in diploid and tetraploid samples. Splitting the *Arabidopsis* genome into 450 approximately equal (1 cM) chromosome intervals, we observed that 67–69 % of intervals in either diploid or tetraploid genomes contained at least one stringently filtered SNP marker, with the pre-designed goal for the genome to be covered by no less than one SNP marker every cM. With 2.0 M 2 × 100 bp paired-end reads per diploid or tetraploid potato sample, 11,018 genomic fragments were bioinformatically predicted to be sequenced. Experimentally, 1.45–1.60 Mbp (72.5–80.0 %) regions were uniquely mapped by at least 2 sequence reads in each diploid and tetraploid potato sample sequenced, respectively, while deep coverage by no less than 10 reads was achieved for 1.27–1.52 Mbp (63.5–76 %) regions in each sequenced sample. From the uniquely mapped sequence reads, 1779 and 5045 polymorphic markers were identified by stringent screening criteria in diploid and tetraploid samples, respectively. By dividing the 725 Mbp potato genome into 725 almost equal intervals of 1 Mbp, we observed the majority of intervals in diploid (62 %) and tetraploid (87 %) genomes contained at least one such high quality polymorphic marker. The distribution of genetic variants detected from the present potato RAD-seq data covered the whole genome much more uniformly when compared with previously published genotyping-by-sequencing analysis using sequence capture (Uitdewilligen et al. 2013).

Use of sequence reads to determine genotype of an individual shares a common issue of balanced presentation of all relevant alleles at a given site, particularly in polyploid species. This issue becomes a significant concern whenever sequencing depth is low, and an effective way to ease the problem in tetraploids is to increase the sequencing depth (Uitdewilligen et al. 2013). On the other, genotype calling in strict sense should include information of two aspects, allele constituent and dosage of the alleles. In that aspect, allele dosage must be predicted for an individual genotype. Biallelic SNP markers observed from sequence reads are informative for diploid genotypes, but are only partially informative for tetraploid genotypes. For tetraploids, genotype calling in the strict sense from sequence reads must involve dosage diagnosis as attempted by a generic statistical method is yet available and needs to be developed to fill this gap in sequence based genotyping in tetraploid species.

Another important property of the optimized RAD-seq approach as a new method for population genotyping is the consistency of identified sequence based markers among different individuals of the population in question. The high quality SNP markers reported here are shared by at least 80 % of individuals in the diploid or tetraploid Arabidopsis or potato populations. Pooling of DNA samples is an effective way to reduce the costs of sequencing library construction. However, sequencing pools with a large number of samples may inherently cause large variation in the number of sequence reads allocated to the constituent samples. Although a larger number of samples were pooled in previous RAD-seq experiments (Andolfatto et al. 2011; Elshire et al. 2011), we proposed here to use 12 samples in every DNA sample pool for sequencing library construction. This, together with use of Qubit (Life Technology, USA) for DNA quantification, confers a good balance between the library construction cost and uniformity in presentation of samples in the pool.

### **Author contribution statement**

ZL and DW conceived and designed the experiments. NJ, FZ, JW, YC and XH performed the experiments. NJ, YC and LL analyzed the data. NJ and XH contributed reagents/materials/analysis tools. ZL, NJ and LL wrote the manuscript.

## Electronic supplementary material

Below is the link to the electronic supplementary material.

S1 Figure. Distribution of selected DNA fragments across the *Arabidopsis* (A) and potato (B) genomes. The black bars below each chromosome indicate the centromere regions (PDF 459 kb)

S2 Figure. Distribution of detectable regions across the potato genome from a published hybrid capture sequencing study (Uitdewilligen et al. 2013) (PDF 46 kb)

Supplementary material 3 (PDF 72 kb)

Supplementary material 4 (DOCX 49 kb)
