# Supplementary figures and images for: A highly robust and optimized sequence-based approach for genetic polymorphism discovery and genotyping in large plant populations
Source: Theor Appl Genet. 2016 Jun 17;129:1739–57. doi: 10.1007/s00122-016-2736-9 (PMC4983294; doi:10.1007/s00122-016-2736-9)

a) in *Arabidopsis* genome

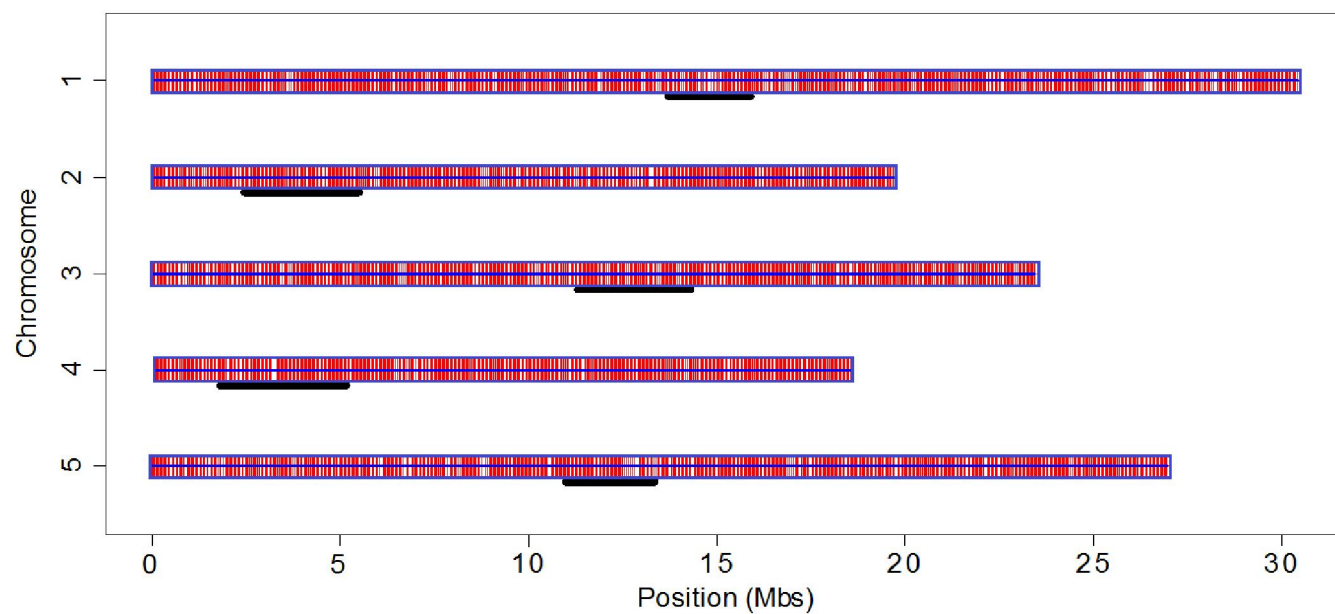

b) in Potato genome

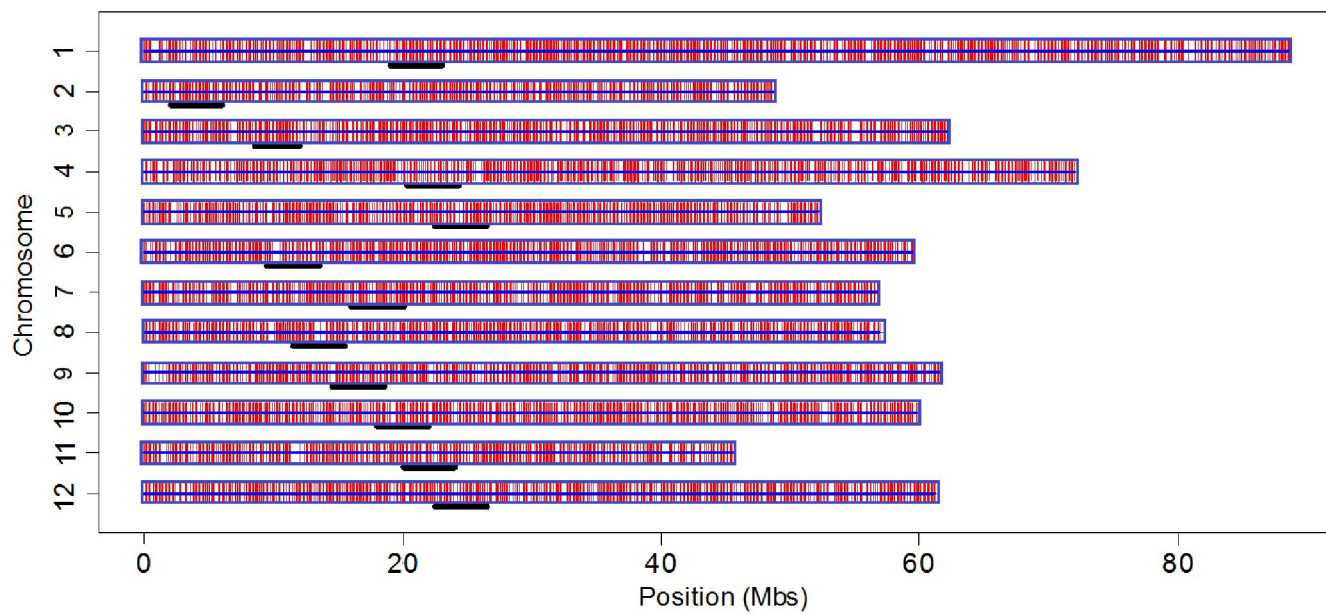

Supplement: Supplementary file 1 — S1 Figure. Distribution of selected DNA fragments across the Arabidopsis (A) and potato (B) genomes. The black bars below each chromosome indicate the centromere regions (PDF 459 kb) [file 122_2016_2736_MOESM1_ESM.pdf]

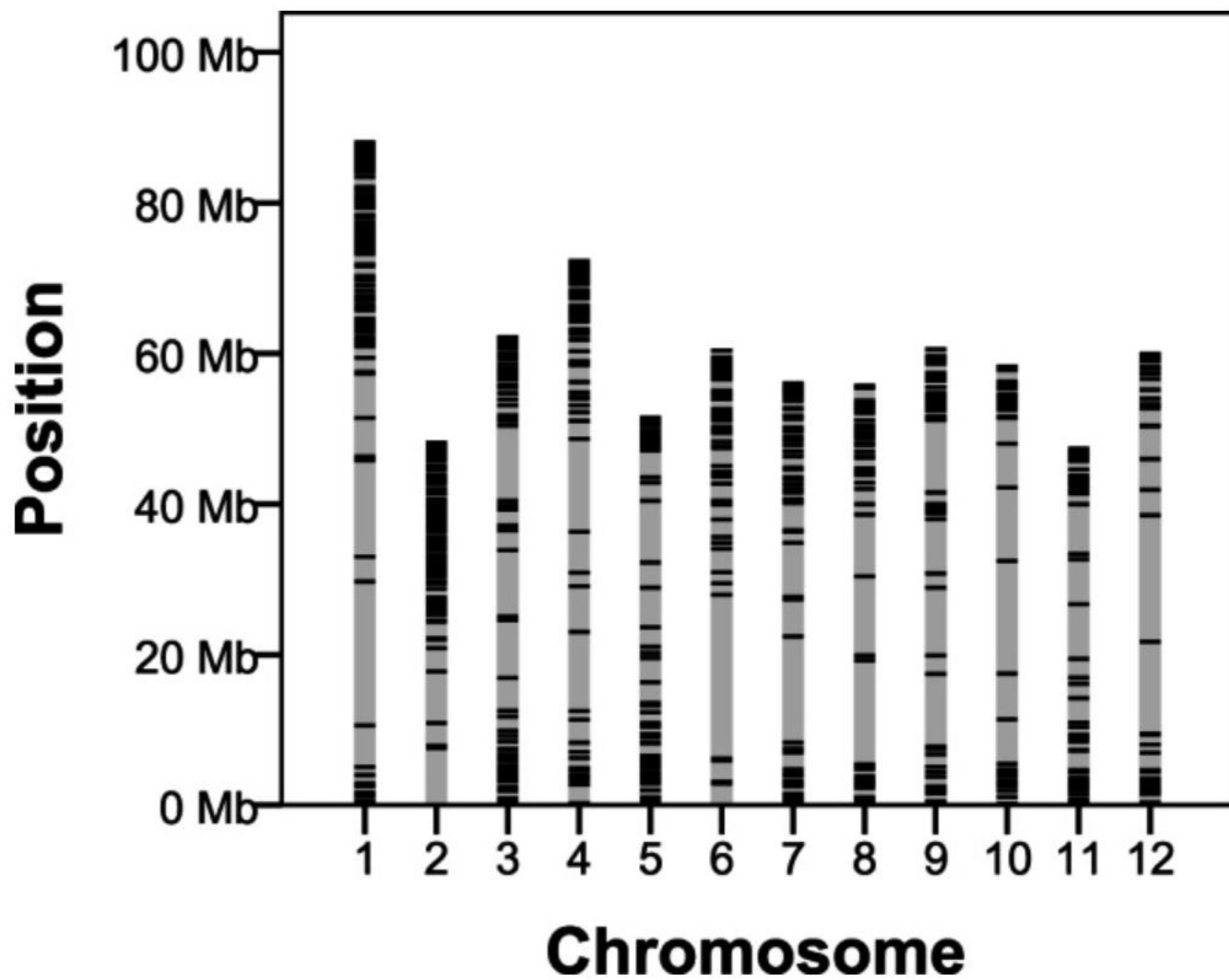

Supplement: Supplementary file 2 — S2 Figure. Distribution of detectable regions across the potato genome from a published hybrid capture sequencing study (Uitdewilligen et al. 2013) (PDF 46 kb) [file 122_2016_2736_MOESM2_ESM.pdf]
