# Supplementary material for: A highly robust and optimized sequence-based approach for genetic polymorphism discovery and genotyping in large plant populations
Source: Theor Appl Genet. 2016 Jun 17;129:1739–57. doi: 10.1007/s00122-016-2736-9 (PMC4983294; doi:10.1007/s00122-016-2736-9)
Supplement: Supplementary file 3 — Supplementary material 3 (PDF 72 kb) [file 122_2016_2736_MOESM3_ESM.pdf]

Modified RAD-seq protocol

First round of digestion to shear cellular DNA

1. Prepare intact, high-quality cellular DNA samples each containing 500ng at a high concentration (≥20ng/ul).
2. Prepare a digestion master mix. The following recipe is for a single reaction, thus multiply by the number of samples plus a small allowance for pipetting error.

10X CutSmart buffer:	5ul
Restriction enzymes combination:	
For Arabidopsis species, EcoRI-HF (20U/ul)	0.25ul
MspI (20U/ul)	0.25ul
HindIII (20U/ul)	0.25ul
For potato species, EcoRI-HF (20U/ul)	0.25ul
MspI (20U/ul)	0.25ul

(NOTE: the RE combination can be changed based on different experimental objectives.)

3. Combine 5.50ul digestion master mix with 500ng DNA sample. Then, add proper amount of H_2O to obtain a total reaction volume of 50ul. Incubate at 37℃ for 3 hours, then hold at 4℃.
4. Clean the digestion reaction system with AMPure XP beads following the standard protocol into a final volume of 30ul for ligation. (NOTE: we have had good success using 1.5X volume of AMPure XP beads to clean the digestion reaction system, and recommend this ratio for all cleaning steps.)
5. Quantify the concentration of cleaned digests. Using Qubit (Invitrogen) to measure DNA concentration is highly recommended; these measurements will be used for preparing ligations.

Adapter Ligation

1. Prepare the following master mix for ligations. This recipe is only for a single reaction, so multiply by the number of samples plus a small allowance for pipetting error.

10X T4 ligase buffer:	3ul
T4 DNA ligase (400U/ul):	0.5ul
10uM universal adapter (MspI adapter):	0.5ul

2. Combine 300ng digested DNA and 0.5ul 10uM barcoded adapter(s) with 4ul ligation master mix. Then, add a proper amount of H_2O to obtain a total reaction volume of 30ul. Incubate at room temperature (23℃) for 2 hours.

Sample pooling and first round of size selection

1. Samples individually ligated with unique-barcoded adapters can be pooled. Here, we pool 12 samples together for further RAD-seq library construction.
2. Clean the pooled sample ligation reaction system following the standard AMPure XP beads protocol using a 1.5x ratio of bead solution, eluting into a final volume of 30ul for size selection.
3. Size selection following the standard Sage Science Pippin-Prep size-selection protocol. (NOTE: when specifying a range for size selection, it is crucial to account for the additional 76 bps of adapter DNA added in ligation.) For our experimental design, we want to select the DNA fragments with the size range from 224 bps to 424 bps. Thus, the target size range should be specified from 300 bps to 500 bps. The Sage Science Pippin-Prep will finally concentrate the targeted DNA fragments into 40ul elution buffer.

Second round of digestion to remove the DNA fragments derived from chloroplast sequence and rRNA genes

1. Prepare a digestion master mix. The following recipe is for a single reaction, thus multiply by the number of pooled libraries plus a small allowance for pipetting error.

10X Tango buffer:	5ul
Restriction enzymes combination:	
For Arabidopsis species, TfiI (20U/ul)	0. 5ul
SnaBI (20U/ul)	0. 5ul
StuI (20U/ul)	0. 5ul
For potato species, HinfI (20U/ul)	0. 5ul
HhaI (20U/ul)	0. 5ul

(NOTE: the RE combination can be changed based on different experimental objectives.)

2. Combine 6ul digestion master mix with 40ul size selected samples. Then, add a proper amount of H_2O to obtain a total reaction volume of 50ul. Incubate at 37°C for 3 hours, then hold at 4°C.
3. Clean the digestion reaction system with AMPure XP beads following the standard protocol into a final volume of 30ul.
4. Clean up with Streptavidin Dynabeads following the standard Invitrogen protocol for both streptavidin bead preparation and hybridization of beads with biotin-labeled DNA, eluting in 20ul. Although only fragments ligated to both universal and barcoded adapters will cluster during sequencing, fragments with barcoded adapters on both 5’ and 3’ ends will still amplify in the subsequent PCR reaction. If the final sequencing library contains many fragments with two barcoded adapter ends, then it becomes difficult to quantify how much sample to load onto the Illumina sequencer for optimal cluster generation. Fragments with both barcoded adapter ends can be removed from libraries using Streptavidin Dynabeads and biotin-labeled universal adapter.
5. Clean the Streptavidin Dynabeads elution system with AMPure XP beads following the standard protocol into a final volume of 20ul.

PCR amplification

1. Prepare a PCR master mix. The following recipe is for a single reaction, thus multiply by the number of pooled libraries plus a small allowance for pipetting error.

2X NEBNext$^{@}$ High-Fidelity PCR Master Mix:	15ul
25uM illumina PCR1 primer:	0.2ul
25uM illumina PCR2 primer:	0.2ul

2. Combine 15.4ul PCR master mix with 10ul DNA samples. Then, add appropriate H_2O to obtain a total reaction volume of 30ul.
3. Run 13-15 cycles of PCR using the following conditions:
    a. Choose the thermal cycler pre-heat lid option and set to 100°C
    b. 98°C for 30 seconds
    c. 13-15 cycles of:
        – 98°C for 30 seconds
        – 56°C for 30 seconds

   – 72℃ for 30 seconds

d. 72℃ for 30 seconds

e. 4℃ hold

Second round of size selection

1. Clean the PCR reaction system with AMPure XP beads following the standard protocol, eluting in 40ul.
2. Size selection following the standard Sage Science Pippin-Prep size-selection protocol. In this step, the targeted DNA fragments are purified to exclude any incorrect constructs that may emerge during the PCR reaction (for example, primer dimers). (NOTE: when specifying the range for size selection, it is crucial to account for the additional 136 bps of Illumina PCR primers added during the PCR reaction.) For our experimental design, we want to select the DNA fragments with the size range from 224 bps to 424 bps. Thus, the target size range should be specified as 360 bps to 560 bps. The Sage Science Pippin-Prep will finally concentrate the targeted DNA fragments into 40ul elution buffer.
3. Clean the size-selection elution system with AMPure XP beads following the standard protocol, eluting in 20ul. This material is now ready for sequencing. (NOTE: Using Qubit (Invitrogen) to measure DNA concentration is highly recommended.)
