# Supplementary material for: A highly robust and optimized sequence-based approach for genetic polymorphism discovery and genotyping in large plant populations
Source: Theor Appl Genet. 2016 Jun 17;129:1739–57. doi: 10.1007/s00122-016-2736-9 (PMC4983294; doi:10.1007/s00122-016-2736-9)
Supplement: Supplementary file 4 — Supplementary material 4 (DOCX 49 kb) [file 122_2016_2736_MOESM4_ESM.docx]

S1 Table. A complete list of all Illumina adapters used in the optimized RAD-seq study.

a) Sample-specific adapters for EcoRI cut site ligation

| Adapters | Nucleotide sequence |
| --- | --- |
| ACTGG_EcoRI_P1.1 | ACACTCTTTCCCTACACGACGCTCTTCCGATCTACTGG |
| AGCTA_EcoRI_P1.2 | ACACTCTTTCCCTACACGACGCTCTTCCGATCTAGCTA |
| ATACG_EcoRI_P1.3 | ACACTCTTTCCCTACACGACGCTCTTCCGATCTATACG |
| CGATC_EcoRI_P1.4 | ACACTCTTTCCCTACACGACGCTCTTCCGATCTCGATC |
| CGTAC_EcoRI_P1.5 | ACACTCTTTCCCTACACGACGCTCTTCCGATCTCGTAC |
| CTGAT_EcoRI_P1.6 | ACACTCTTTCCCTACACGACGCTCTTCCGATCTCTGAT |
| GAGTC_EcoRI_P1.7 | ACACTCTTTCCCTACACGACGCTCTTCCGATCTGAGTC |
| GCTGA_EcoRI_P1.8 | ACACTCTTTCCCTACACGACGCTCTTCCGATCTGCTGA |
| GTCGA_EcoRI_P1.9 | ACACTCTTTCCCTACACGACGCTCTTCCGATCTGTCGA |
| TACCG_EcoRI_P1.10 | ACACTCTTTCCCTACACGACGCTCTTCCGATCTTACCG |
| GCATG_EcoRI_P1.11 | ACACTCTTTCCCTACACGACGCTCTTCCGATCTGCATG |
| TCAGT_EcoRI_P1.12 | ACACTCTTTCCCTACACGACGCTCTTCCGATCTTCAGT |
| ACTGG_EcoRI_P2.1 | [Phos]AATTCCAGTAGATCGGAAGAGCGTCGTGTAGGGAAAGAGTGT |
| AGCTA_EcoRI_P2.2 | [Phos]AATTTAGCTAGATCGGAAGAGCGTCGTGTAGGGAAAGAGTGT |
| ATACG_EcoRI_P2.3 | [Phos]AATTCGTATAGATCGGAAGAGCGTCGTGTAGGGAAAGAGTGT |
| CGATC_EcoRI_P2.4 | [Phos]AATTGATCGAGATCGGAAGAGCGTCGTGTAGGGAAAGAGTGT |
| CGTAC_EcoRI_P2.5 | [Phos]AATTGTACGAGATCGGAAGAGCGTCGTGTAGGGAAAGAGTGT |
| CTGAT_EcoRI_P2.6 | [Phos]AATTATCAGAGATCGGAAGAGCGTCGTGTAGGGAAAGAGTGT |
| GAGTC_EcoRI_P2.7 | [Phos]AATTGACTCAGATCGGAAGAGCGTCGTGTAGGGAAAGAGTGT |
| GCTGA_EcoRI_P2.8 | [Phos]AATTTCAGCAGATCGGAAGAGCGTCGTGTAGGGAAAGAGTGT |
| GTCGA_EcoRI_P2.9 | [Phos]AATTTCGACAGATCGGAAGAGCGTCGTGTAGGGAAAGAGTGT |
| TACCG_EcoRI_P2.10 | [Phos]AATTCGGTAAGATCGGAAGAGCGTCGTGTAGGGAAAGAGTGT |
| GCATG_EcoRI_P2.11 | [Phos]AATTCATGCAGATCGGAAGAGCGTCGTGTAGGGAAAGAGTGT |
| TCAGT_EcoRI_P2.12 | [Phos]AATTACTGAAGATCGGAAGAGCGTCGTGTAGGGAAAGAGTGT |

The unique 5bp barcodes are highlighted in yellow.

b) Sample-specific adapters for HindIII cut site ligation.

| Adapters | Nucleotide sequence |
| --- | --- |
| ACTGG_HindIII_P1.1 | ACACTCTTTCCCTACACGACGCTCTTCCGATCTACTGG |
| AGCTA_ HindIII _P1.2 | ACACTCTTTCCCTACACGACGCTCTTCCGATCTAGCTA |
| ATACG_ HindIII _P1.3 | ACACTCTTTCCCTACACGACGCTCTTCCGATCTATACG |
| CGATC_ HindIII _P1.4 | ACACTCTTTCCCTACACGACGCTCTTCCGATCTCGATC |
| CGTAC_ HindIII _P1.5 | ACACTCTTTCCCTACACGACGCTCTTCCGATCTCGTAC |
| CTGAT_ HindIII _P1.6 | ACACTCTTTCCCTACACGACGCTCTTCCGATCTCTGAT |
| GAGTC_ HindIII _P1.7 | ACACTCTTTCCCTACACGACGCTCTTCCGATCTGAGTC |
| GCTGA_ HindIII _P1.8 | ACACTCTTTCCCTACACGACGCTCTTCCGATCTGCTGA |
| GTCGA_ HindIII _P1.9 | ACACTCTTTCCCTACACGACGCTCTTCCGATCTGTCGA |
| TACCG_ HindIII _P1.10 | ACACTCTTTCCCTACACGACGCTCTTCCGATCTTACCG |
| GCATG_ HindIII _P1.11 | ACACTCTTTCCCTACACGACGCTCTTCCGATCTGCATG |
| TCAGT_ HindIII _P1.12 | ACACTCTTTCCCTACACGACGCTCTTCCGATCTTCAGT |
| ACTGG_ HindIII _P2.1 | [Phos]AGCTCCAGTAGATCGGAAGAGCGTCGTGTAGGGAAAGAGTGT |
| AGCTA_ HindIII _P2.2 | [Phos]AGCTTAGCTAGATCGGAAGAGCGTCGTGTAGGGAAAGAGTGT |
| ATACG_HindIII_P2.3 | [Phos]AGCTCGTATAGATCGGAAGAGCGTCGTGTAGGGAAAGAGTGT |
| CGATC_HindIII_P2.4 | [Phos]AGCTGATCGAGATCGGAAGAGCGTCGTGTAGGGAAAGAGTGT |
| CGTAC_HindIII_P2.5 | [Phos]AGCTGTACGAGATCGGAAGAGCGTCGTGTAGGGAAAGAGTGT |
| CTGAT_HindIII_P2.6 | [Phos]AGCTATCAGAGATCGGAAGAGCGTCGTGTAGGGAAAGAGTGT |
| GAGTC_HindIII_P2.7 | [Phos]AGCTGACTCAGATCGGAAGAGCGTCGTGTAGGGAAAGAGTGT |
| GCTGA_HindIII_P2.8 | [Phos]AGCTTCAGCAGATCGGAAGAGCGTCGTGTAGGGAAAGAGTGT |
| GTCGA_HindIII_P2.9 | [Phos]AGCTTCGACAGATCGGAAGAGCGTCGTGTAGGGAAAGAGTGT |
| TACCG_HindIII_P2.10 | [Phos]AGCTCGGTAAGATCGGAAGAGCGTCGTGTAGGGAAAGAGTGT |
| GCATG_HindIII_P2.11 | [Phos]AGCTCATGCAGATCGGAAGAGCGTCGTGTAGGGAAAGAGTGT |
| TCAGT_HindIII_P2.12 | [Phos]AGCTACTGAAGATCGGAAGAGCGTCGTGTAGGGAAAGAGTGT |

The unique 5bp barcodes are highlighted in yellow.

c) Universal adapters for MspI cut site ligation

| Adapters | Nucleotide sequence |
| --- | --- |
| MspI_P1 | [Biotin]GTGACTGGAGTTCAGACGTGTGCTCTTCCGATCT |
| MspI_P2 | [Phos]CGAGATCGGAAGAGCGAGAACAA |

S2 Table. Copy number of chloroplast DNA in leaf tissue across different plant species.

| Plant species | Copy number of chloroplast sequence per cell | Reference |
| --- | --- | --- |
| *Arabidopsis* | 1,000 ~ 1,500 | 1 |
| Barley | 2,000 to more than 8,000 | 2 |
| Beta *vulgaris* | 1,100 ~ 1,900 | 3 |
| spinach | 3,900 ~ 5,100 | 4 |
| Pea | 6,000 ~ 9,000 | 5 |
| Medicago *truncatula* | 2,600 ~ 19,000 | 6 |
| wheat | 40,000 ~ 50,000 | 7 |
| Tobacco | approximately 10,000 | 6 |

References

1. Zoschke R, Liere K, Börner T (2007) From seedling to mature plant: Arabidopsis plastidial genome copy number, RNA accumulation and transcription are differentially regulated during leaf development. The Plant Journal **50**: 710-722.

2. Baumgartner BJ, Rapp JC, Mullet JE (1989) Plastid Transcription Activity and DNA Copy Number Increase Early in Barley Chloroplast Development. Plant Physiology **89**: 1011-1018.

3. Tymms MJ, Scott NS, Possingham JV (1983) DNA Content of Beta vulgaris Chloroplasts during Leaf Cell Expansion. Plant Physiology **71**: 785-788.

4. Scott NS, Possingham JV (1980) Chloroplast DNA in Expanding Spinach Leaves. Journal of Experimental Botany **31**: 1081-1092.

5. Lamppa GK, Elliot LV, Bendich AJ (1980) Changes in Chloroplast Number during Pea Leaf Development: An Analysis of a Protoplast Population. Planta **148**: 437-443.

6. Shaver J, Oldenburg D, Bendich A (2006) Changes in chloroplast DNA during development in tobacco, Medicago truncatula, pea, and maize. Planta **224**: 72-82.

7. Boffey SA, Leech RM (1982) Chloroplast DNA Levels and the Control of Chloroplast Division in Light-Grown Wheat Leaves. Plant Physiology **69**: 1387-1391.

S3 Table. Distribution of the number of short read pairs allocated to each sample in each pooled sequencing library.

a) *Arabidopsis* 12 sample pooled RAD-seq dataset

| Sample ID | Diploid pooled samples | | | | Tetraploid pooled samples | | |
| --- | --- | --- | --- | --- | --- | --- | --- |
|  | *Read pairs | proportion | | expected | *Read Pairs | proportion | expected |
| F2_1 | 1.04 | 6.93% | | 8.33% | 1.39 | 9.80% | 8.33% |
| F2_2 | 1.51 | 10.04% | | 8.33% | 1.43 | 10.08% | 8.33% |
| F2_3 | 1.14 | 7.56% | | 8.33% | 1.36 | 9.62% | 8.33% |
| F2_4 | 1.30 | 8.61% | | 8.33% | 1.00 | 7.08% | 8.33% |
| F2_5 | 0.91 | 6.00% | | 8.33% | 0.88 | 6.22% | 8.33% |
| F2_6 | 1.65 | 10.98% | | 8.33% | 0.74 | 5.20% | 8.33% |
| F2_7 | 1.05 | 6.95% | | 8.33% | 1.09 | 7.68% | 8.33% |
| F2_8 | 1.05 | 6.97% | | 8.33% | 1.05 | 7.42% | 8.33% |
| F2_9 | 1.07 | 7.13% | | 8.33% | 1.22 | 8.63% | 8.33% |
| F2_10 | 1.42 | 9.44% | | 8.33% | 1.22 | 8.61% | 8.33% |
| P1 | 1.13 | 7.49% | | 8.33% | 1.01 | 7.16% | 8.33% |
| P2 | 1.39 | 9.21% | | 8.33% | 1.32 | 9.30% | 8.33% |
| undetermined | 0.41 | 2.70% | | 0.00% | 0.45 | 3.20% | 0.00% |
| total | 15.08 | 100.00% | | 100.00% | 14.16 | 100.00% | 100.00% |
| Coefficient of variation | | | 18.6% | 0.0% |  | 19.4% | 0.0% |

b) Potato 12 sample pooled RAD-seq dataset

| Sample ID | Diploid pooled samples | | | Tetraploid pooled samples | | | |
| --- | --- | --- | --- | --- | --- | --- | --- |
|  | *Read pairs | proportion | expected | *Read pairs | | proportion | expected |
| F1_1 | 1.93 | 7.68% | 8.33% | 1.99 | | 7.84% | 8.33% |
| F1_2 | 2.01 | 7.96% | 8.33% | 2.31 | | 9.09% | 8.33% |
| F1_3 | 2.05 | 8.14% | 8.33% | 2.00 | | 7.90% | 8.33% |
| F1_4 | 2.38 | 9.45% | 8.33% | 1.91 | | 7.52% | 8.33% |
| F1_5 | 2.25 | 8.92% | 8.33% | 2.02 | | 7.94% | 8.33% |
| F1_6 | 2.04 | 8.08% | 8.33% | 1.94 | | 7.64% | 8.33% |
| F1_7 | 1.58 | 6.29% | 8.33% | 2.16 | | 8.52% | 8.33% |
| F1_8 | 2.14 | 8.51% | 8.33% | 2.18 | | 8.58% | 8.33% |
| F1_9 | 1.96 | 7.76% | 8.33% | 1.93 | | 7.62% | 8.33% |
| F1_10 | 2.24 | 8.88% | 8.33% | 2.17 | | 8.54% | 8.33% |
| P1 | 2.04 | 8.09% | 8.33% | 1.95 | | 7.68% | 8.33% |
| P2 | 2.05 | 8.15% | 8.33% | 2.43 | | 9.59% | 8.33% |
| undetermined | 0.53 | 2.10% | 0.00% | 0.39 | | 1.53% | 0.00% |
| total | 25.19 | 100.00% | 100.00% | 25.37 | | 100.00% | 100.00% |
| Coefficient of variation | | 9.6% | 0.0% |  | 8.0% | | 0.0% |

* millions of reads

S4 Table. The number of short paired-end reads allocated to each of the 6 pooled samples in the sequencing libraries that underwent only the first round of RE digestion.

| Sample ID | *Arabidopsis* pooled samples | | | Sample ID | Potato pooled samples | | |
| --- | --- | --- | --- | --- | --- | --- | --- |
|  | Reads (M) | proportion | expected |  | Reads (M) | proportion | expected |
| Columbia (d)* | 0.53 | 18.68% | 16.67% | BD66-6 | 1.07 | 21.19% | 16.67% |
| Landsberg (d) | 0.40 | 14.29% | 16.67% | BD6-6 | 0.95 | 18.82% | 16.67% |
| F2_1 (d) | 0.32 | 11.42% | 16.67% | F1_1 (d) | 0.54 | 10.67% | 16.67% |
| Columbia (t)** | 0.47 | 16.69% | 16.67% | Atlantic | 0.91 | 18.01% | 16.67% |
| Landsberg (t) | 0.53 | 18.83% | 16.67% | Longsu-3 | 0.96 | 19.04% | 16.67% |
| F2_1 (t) | 0.50 | 17.90% | 16.67% | F1_1 (t) | 0.56 | 11.08% | 16.67% |
| undetermined | 0.06 | 2.17% | 0.00% | undetermined | 0.06 | 1.27% | 0.00% |
| total | 2.82 | 100% | 100.00% | total | 5.06 | 100% | 100.00% |

* (d), diploid

** (t), tetraploid

S5 Table. Coverage of potato RAD-seq reads in Mbp across the genome and in selected genome regions (2.0 Mbp).

| Sample  ID |  | | Diploid pooled samples | | | | | | | | | | | | | |  | | Tetraploid pooled samples | | | | | | | | | | | | | |
| --- | --- | --- | --- | --- | --- | --- | --- | --- | --- | --- | --- | --- | --- | --- | --- | --- | --- | --- | --- | --- | --- | --- | --- | --- | --- | --- | --- | --- | --- | --- | --- | --- |
|  |  |  | Whole genome | | | | | |  | | Selected regions | | | | | |  | | Whole genome | | | | | |  | | Selected regions | | | | | |
|  |  |  | Covered* | |  | | Deep** | |  | | Covered* | |  | | Deep* | |  | | Covered* | |  | | Deep** | |  | | Covered* | |  | | Deep** | |
| F2_1 | |  | | 5.37 | |  | | 2.77 | |  | | 1.51 | |  | | 1.35 | |  | | 4.39 | |  | | 2.25 | |  | | 1.49 | |  | | 1.32 |
| F2_2 | |  | | 5.61 | |  | | 2.94 | |  | | 1.57 | |  | | 1.43 | |  | | 5.32 | |  | | 2.23 | |  | | 1.50 | |  | | 1.30 |
| F2_3 | |  | | 5.42 | |  | | 2.86 | |  | | 1.55 | |  | | 1.40 | |  | | 4.47 | |  | | 2.26 | |  | | 1.52 | |  | | 1.33 |
| F2_4 | |  | | 5.43 | |  | | 2.94 | |  | | 1.50 | |  | | 1.38 | |  | | 4.59 | |  | | 2.19 | |  | | 1.49 | |  | | 1.29 |
| F2_5 | |  | | 5.59 | |  | | 3.02 | |  | | 1.56 | |  | | 1.43 | |  | | 4.54 | |  | | 2.28 | |  | | 1.51 | |  | | 1.33 |
| F2_6 | |  | | 5.03 | |  | | 2.57 | |  | | 1.48 | |  | | 1.32 | |  | | 4.45 | |  | | 2.18 | |  | | 1.45 | |  | | 1.27 |
| F2_7 | |  | | 5.47 | |  | | 2.88 | |  | | 1.56 | |  | | 1.41 | |  | | 4.45 | |  | | 2.26 | |  | | 1.44 | |  | | 1.27 |
| F2_8 | |  | | 5.24 | |  | | 2.86 | |  | | 1.53 | |  | | 1.39 | |  | | 4.50 | |  | | 2.25 | |  | | 1.42 | |  | | 1.27 |
| F2_9 | |  | | 4.80 | |  | | 2.20 | |  | | 1.54 | |  | | 1.34 | |  | | 5.52 | |  | | 2.21 | |  | | 1.53 | |  | | 1.32 |
| F2_10 | |  | | 5.22 | |  | | 2.36 | |  | | 1.60 | |  | | 1.42 | |  | | 4.57 | |  | | 2.30 | |  | | 1.45 | |  | | 1.29 |
| P1 | |  | | 5.57 | |  | | 2.89 | |  | | 1.67 | |  | | 1.52 | |  | | 4.11 | |  | | 2.16 | |  | | 1.45 | |  | | 1.29 |
| P2 | |  | | 5.12 | |  | | 2.73 | |  | | 1.50 | |  | | 1.35 | |  | | 4.59 | |  | | 2.45 | |  | | 1.54 | |  | | 1.38 |

*****at least 2 reads uniquely mapped

******at least 10 reads uniquely mapped

S6 table. Expected genotype frequencies in potato tetraploid F1 offspring populations.

| Genotype in two parents | | | Double-reduction parameter | | | Genotype frequencies in F1 population | | | |
| --- | --- | --- | --- | --- | --- | --- | --- | --- | --- |
| 1 (homozygous) |  | 2 (heterozygous) |  |  |  | homozygous | |  | heterozygous |
| AAAA |  | AAAa |  | α |  | | (2+ α)/4 |  | (2- α)/4 |
| AAAA |  | AAaa |  | α |  | | (1+2 α)/6 |  | (5-2 α)/6 |
| AAAA |  | Aaaa |  | α |  | | α/4 |  | (4- α)/4 |

S7 Table. In silico analysis result of the ‘MseI’ RE’s digestion of grape reference sequences from RAD-seq method (Wang et al. 2012a).

|  | ‘MseI’ RE selected DNA fragment* | | |
| --- | --- | --- | --- |
|  | genome | rRNA | chloroplast |
| Number of selected DNA fragments per haploid genome | 142,655 | 4 | 63 |

***** DNA fragment with length from 300bps to 400bps

Wang N, Fang LC, Xin HP, Wang LJ, Li SH (2012a) Construction of a high-density genetic map for grape using next generation restriction-site associated DNA sequencing. BMC Plant Biology **12**: 148.

S8 Table. Proportion of short sequence reads generated from 2b-RAD method (Wang et al. 2012b) and mapped to genomic, rRNA genes and chloroplast regions of *Arabidopsis* sequence.

1. predicted

| Type IIB RE | BsaXI | | | | | AlfI | | | BsaXI-RTR* | | | |
| --- | --- | --- | --- | --- | --- | --- | --- | --- | --- | --- | --- | --- |
| DNA sequence origin | genome | rRNA | | chloroplast | | genome | rRNA | chloroplast | genome | rRNA | | chloroplast |
| # of DNA fragments per haploid genome | 40,250 | 4 | | 54 | | 12,717 | 0 | 12 | 826 | 0 | | 1 |
| # of copies | 2 | 2x700 | | 1,200 | | 2 | 2x700 | 1,200 | 2 | 2x700 | | 1,200 |
| # of selected DNA fragments per cell | 80,500 | 5,600 | | 64,800 | | 25,434 | 0 | 14,400 | 1,652 | 0 | | 1,200 |
| # of selected DNA fragments per cell | 150,900 | | | | | 39,834 | | | 2,852 | | | |
| % of reads mapped to different regions | **53.3** | | 3.7 | | **42.9** | 63.8 | 0 | 36.2 | **57.9** | | 0 | 42.1 |

*Reduced tag representation library

1. observed

| Material & REs  Sequencing Platform | TxK* BsaXI Illumina |  | TxK* BsaXI AB SOLiD |  | Ler BsaXI  AB SOLiD |  | Ler AlfI  AB SOLiD |  | Ler BsaXI-RTR  AB SOLiD |
| --- | --- | --- | --- | --- | --- | --- | --- | --- | --- |
| Unmapped | 42 |  | 58 |  | 40 |  | 61 |  | 52 |
| Mapped | **58** |  | **42** |  | **60** |  | **39** |  | **48** |
| genomic | 64 |  | 61 |  | 62 |  | 67 |  | 70 |
| non-genomic** | 36 |  | 39 |  | 38 |  | 33 |  | 30 |

* TxK -- Tsu-1 x Kas-1 F1 individual

Wang S, Meyer E, McKay JK, Matz MV (2012b) 2b-RAD-seq: a simple and flexible method for genome-wide genotyping. Nat Meth **9**: 808.
